# Cynomolgus macaque IL37 polymorphism and control of SIV infection

**DOI:** 10.1038/s41598-019-44235-x

**Published:** 2019-05-28

**Authors:** Takashi Shiina, Shingo Suzuki, Nicolas Congy-Jolivet, Alice Aarnink, Henri-Jean Garchon, Nathalie Dereuddre-Bosquet, Bruno Vaslin, Nicolas Tchitchek, Delphine Desjardins, Brigitte Autran, Olivier Lambotte, Ioannis Theodorou, Roger Le Grand, Antoine Blancher

**Affiliations:** 1 Department of Molecular Life Sciences, Division of Basic Medical Science and Molecular Medicine, Tokai University School of Medicine, 143 Shimokasuya, Isehara, Kanagawa 259-1193 Japan; 2 Laboratoire d’immunogénétique moléculaire (LIMT, EA 3034, Faculté de médecine Purpan, Université Toulouse 3, Paul Sabatier, UPS), Toulouse, France; 3 Laboratoire d’immunologie, CHU de Toulouse, Institut Fédératif de Biologie, hôpital Purpan, 330 Avenue de Grande Bretagne, TSA40031, 31059 Toulouse, cedex 9 France; 4 Inserm U1173, Simone Veil School of Health Sciences, University of Versailles Saint-Quentin-en-Yvelines, Montigny-le-Bretonneux, France; 5 Genetics Division, Ambroise Paré Hospital (AP-HP), Boulogne-Billancourt, France; 6 CEA – Université Paris-Sud 11 – INSERM U1184, Immunology of Viral Infections and Autoimmune Diseases, IDMIT Department, IBFJ, 92265 Fontenay-aux-Roses, France; 7 U1135, CIMI, INSERM, Paris, France, Sorbonne Universités, UPMC Université Paris 06, Paris, France, Département d’Immunologie, Hôpital Pitié-Salpêtrière, AP-HP, Paris, France; 8 Assistance Publique – Hôpitaux de Paris, Service de Médecine Interne et Immunologie Clinique, Groupe Hospitalier Universitaire Paris Sud, Hôpital Bicêtre, Le Kremlin‐Bicêtre, France, Université Paris Sud, Le Kremlin Bicêtre, France; 9 Center for Immunology and Infectious Diseases, INSERM UMR S 1135, Pierre et Marie Curie University, Paris, France; 10 Centre de Physiopathologie Toulouse-Purpan (CPTP), Université de Toulouse, Centre National de la Recherche Scientifique (CNRS), Institut National de la Santé et de la Recherche Médicale (Inserm), Université Paul Sabatier (UPS), Toulouse, France

**Keywords:** Immunogenetics, Immunogenetics, HIV infections, HIV infections

## Abstract

The association between gene polymorphisms and plasma virus load at the set point (SP-PVL) was investigated in Mauritian macaques inoculated with SIV. Among 44 macaques inoculated with 50 AID50, six individuals were selected: three with SP-PVL among the highest and three with SP-PVL among the lowest. The exons of 390 candidate genes of these six animals were sequenced. Twelve non-synonymous single nucleotide polymorphisms (NS-SNPs) lying in nine genes potentially associated with PVL were genotyped in 23 animals. Three NS-SNPs with probabilities of association with PVL less than 0.05 were genotyped in a total of 44 animals. One NS-SNP lying in exon 1 of the IL37 gene displayed a significant association (*p* = 3.33 × 10^−4^) and a strong odds ratio (19.52). Multiple linear regression modeling revealed three significant predictors of SP-PVL, including the IL37 exon 1 NS-SNP (*p* = 0.0004) and the MHC Class IB haplotypes M2 (*p* = 0.0007) and M6 (*p* = 0.0013). These three factors in conjunction explained 48% of the PVL variance (*p* = 4.8 × 10^−6^). The potential role of IL37 in the control of SIV infection is discussed.

## Introduction

Among the Old World monkeys used as animal models of SIV infection, the cynomolgus macaque (*Macaca fascicularis*, hereinafter abbreviated as *Mafa*), which is easy to breed in captivity, is increasingly being used^1^. The geographic area of *Mafa* in Southwest Asia is very large, and genetic studies, especially the most recent ones based on genome-wide studies, have revealed strong genetic differentiation between different natural populations^2,3^. Five hundred years ago, a small number of animals captured in Indonesia (Java and/or Sumatra) and Malaysia were released in Mauritius and led to the establishment of an artificial population^4,5^. Due to the high bottleneck and total genetic isolation, the polymorphism of the Mauritian macaque population is significantly reduced compared to natural populations^6–8^. For example, the genetic diversity of the Mauritius cynomolgus macaque population was estimated to be 20% less than that of the Indonesian–Malaysian population^7^. The genetic singularities of the Mauritian macaque population are advantageous for genetic association studies.

Several studies have shown that control of SIV infection in Mauritian cynomolgus macaque was associated with major histocompatibility complex (MHC) polymorphism^9–13^. We previously reported that MHC class IB M2 and M6 haplotypes were associated with low PVL values at the set point (SP-PVL)^10^. A multiple linear regression model showed that MHC class IB polymorphism explained 35% of SP-PVL variance, suggesting that 65% of this variance may be dependent on environmental factors or genetic factors other than MHC. In a previous study, Ericsen *et al*.^14^ identified candidate loci associated to the control of SIV infection, by whole genome sequencing of twelve Mauritian cynomolgus macaques that exhibited highly differing SP-PVL. More recently, we reported on a whole genome association study based on nine animals followed by the assessment of best candidate SNPs on a group of 42 animals^15^.

We present here a different approach based on a candidate gene association study. We explored the association between SIV plasma viral load at the set point (SP-PVL) and polymorphism in the coding regions of 390 candidate genes involved in the progression of viral replication or the immune defense against infection. The polymorphism of these genes was characterized by targeted resequencing of DNA fragments encompassing all exons of the selected candidate genes. The DNA fragments were enriched by means of a customized exon-capture system. In step one, the exonic sequences of six animals were determined using next generation sequencing (NGS). The six animals were selected from a group of 44 animals that were inoculated with 50 AID50 of SIV and did not receive any preventative or curative treatment. Three out of the six selected animals presented SP-PVL among the lowest of the 44 subjects, while the three others had SP-PVL among the highest. This first step allowed us to characterize twelve NS-SNP candidates associated with the PVL. In step two, the number of animals genotyped to determine the three best candidate SNPs was increased to 44. The strong association between the SP-PVL and an NS-SNP lying in the exon 1 of IL37 was confirmed. We discussed the potential impact of IL37 polymorphism in the control of SIV infection in cynomolgus macaques.

## Materials and Methods

### Ethics statement

The DNA samples used in this study were from animals used in control groups of previous SIV infection therapeutic trials, so that no samples were collected specifically for the current study^10^. All the experiments performed on the macaques were carried out in accordance with the directives and regulations in force. Animals were handled in accordance with European guidelines for NHP care (EU Directive N 63/2010) and complying Standards for Human Care and Use of Laboratory Animals of the Office for Laboratory Animal Welfare (OLAW, USA). The CEA is registered under OLAW Assurance number A5826-01. The Ethical Animal Committee of the CEA (“Comité d’Ethique en Expérimentation Animale du Commisariat à l′énergie atomique”), registered by the French Research Ministry under number 44, approved and accredited this study (statement number 12-006).

### Animals

Forty-four male cynomolgus macaques imported from Mauritius (Noveprim, Bioprim) were inoculated either by intravenous (IV, N = 30) or intrarectal (IR, N = 14) injection of 50 AID50 (50 times the 50% animal infectious dose) of the pathogenic SIVmac251 isolate. As detailed by Aarnink *et al*.^10^, the animals received no treatment before or after SIV inoculation. The animals are described in Supplementary Table S1. It should be noted that all but two animals (# 14468 and # 20483) were identical to those described in our previous study^15^.

### Determination of plasma viral load

Plasma viral load (PVL) was measured by quantitative RT-PCR as described by Karlsson *et al*.^16^. The PVL measured around 100 days (range 95–128) after SIV inoculation, corresponded to the set-point of viremia. As SP-PVL followed a log-normal distribution, the logarithm of SP-PVL (log SP-PVL) was used in all calculations. Three animals that showed the highest SP-PVL values (log SP-PVL: 5.2, 5.4 and 5.6) and three animals that showed the lowest SP-PVL values (log SP-PVL: 1.8) were selected for exon capture and target sequencing (see Supplementary Table S1 for details).

### DNA extraction and MHC genotyping

As previously described^10^, genomic DNA was extracted from peripheral blood by using either the QIAamp DNA Blood mini Kit (Qiagen, Courtaboeuf, France) or a standard phenol-chloroform method. The study of 20 microsatellites scattered across the MHC region allowed to deduce the MHC genotypes of the macaques^10^. The MHC DRB genotyping was performed by direct sequencing of exon 2 amplified fragments separated by denaturing gradient gel electrophoresis (DGGE), as previously described by Blancher *et al*.^17^. The MHC haplotypes in the MHC class IB region were deduced by using four microsatellites (B2; PO3; MICA; D6S2793). In Mauritius, there are seven MHC haplotypes which can be defined unambiguously in the MHC class IB region by means of these four microsatellites. In this region, forty-one animals had unambiguous MHC genotypes and three animals, not taken into account in statistics, displayed genotypes suggesting the presence of recombinant haplotypes. The MHC class IB genotypes of the 44 animals are listed in Supplementary Table S1.

### Exon capture and sequencing of six animals

Selection of 390 candidate genes was based on the literature concerning genes involved in HIV replication, GWAS in humans, and experimental data in animal models (see Supplementary Table S2). A custom DNA solution-capture (SeqCap EZ, Nimblegen/Roche) for enriching a total of 681,768 bp (3,883 exons) was designed according to the manufacturer’s recommendation.

Genomic DNA samples from six animals (three with among the highest and three with among the lowest SP-PVL) were studied. For each animal, a DNA library was prepared from one microgram of sonicated genomic DNA sample (M220 Focused ultrasonicator, Covaris, Woburn, MA). Libraries barcoding was performed according to the manufacturer’s protocol (Life Technologies/Thermo Fisher Scientific, Palo Alto, CA). After an eight cycles of PCR amplification, library quality and concentration was determined using the Agilent 2100 Expert Bioanalyzer and the Agilent High Sensitivity DNA Kit (Agilent Technologies, Santa Clara, CA). The six barcoded libraries were mixed at equimolar concentrations before to be submitted to the custom DNA solution-capture (SeqCap EZ, Nimblegen/Roche) according to the manufacturer’s protocol. The library pool enriched in fragments of interest was submitted to an emulsion PCR (emPCR) by using the Ion PGM Template OT2 200 Kit on an Ion OneTouch 2 automated system (Life Technologies). Beads carrying single-stranded DNA templates were enriched according to the manufacturer’s recommendation. Library pool sequencing was performed by using the Ion PGM Sequencing 200 Kit and Ion 318 Chip (flow number of 850 for 200 base-read).

### SNP calling

Sequence reads were binned by the Ion Xpress Barcodes into six separate sequence fastq files using the Torrent Suite 4.2.1 software (Life Technologies/Thermo Fisher Scientific). Reads ending by poor quality sequences (quality values (QVs) less than 10) were remove from the separated sequence files by further trimming. Draft read numbers totalled 16,925,342 sequence reads with reads per animal ranging from 1,685,914 to 7,028,167 (of the high-quality sequence reads) with average quality values (QV) of 23.8 ± 0.3. The draft read bases totaled 2.1 Gb, with an overall average read length of 123.8 ± 5.4 bases. An observed average depth and coverage was 104.0 ± 14.0 from 86.3 to 132.7 and 98.0% ± 0.9 from 97.3% to 100%, respectively (Supplementary Table S3). Therefore, the sequence reads were of high quality and sufficient sequence volume for further polymorphism analysis.

Mapping of the sequence reads on the rhesus monkey complete genome (version 2 as reference) were automatically performed using the GS Reference Mapper Ver. 3.0 software (Roche), and variant databases were generated in each barcode. The mapping parameter was set to a 95% match between the read and reference sequences. After mapping, single nucleotide polymorphisms (SNPs) that distinguished the three high-PVL animals from the three low-PVL animals were extracted from the variant databases generated from the six animals.

### Calculations of odds ratio, probabilities of association

Statistical differences between the two groups separated by the median of log SP-PVL were calculated using the Student’s t-test, performed in Microsoft Excel using the “TTEST” function. The log SP-PVL levels were categorized as “high” or “low” based on the Levene F-test and a median cut-off value of 3.14 for the relative log SP-PVL level. Odds ratios and statistical probabilities were calculated using the “R” program (http://www.r-project.org/), and statistical probabilities were calculated using the Exact Fisher test within the “R” program.

### Multiple linear regressions (MLR)

Multiple linear regressions were used to test the combined influence of genetic polymorphisms on log SP-PVL. Multivariate linear models were studied with the “lm” function in the “R” environment. The genotypes of the SNP were translated into numerical values (0: animals not possessing the variant, 1: heterozygous individuals and 2 animals homozygous for the variant). Likewise, the presence/absence of the seven MHC class IB haplotypes was converted into numerical variables (absence: 0; presence in simple dose: (1) presence in double dose: (2) Using the Akaike information criterion, a step-by-step procedure (forward or backward) was used to select the best multivariate linear model. For the best model, we report the *p* value of the model, the *p* values of significant predictors (ANOVA) and the adjusted R-squared coefficient as an indication of the fraction of variance explained by the predictors of the model.

### SNP genotyping by Sanger sequencing

Validation of the candidate SNP was performed with newly designed, locus-specific primers. In brief, the 20 μL amplification reaction volume contained 10 ng of genomic DNA, comprising KOD FX polymerase (TOYOBO, Osaka, Japan) 2 × PCR buffer, 2 mM of each dNTP and 0.5 μM of each primer (primer sequences are listed in Supplementary Table S4). The cycling parameters were as follows: an initial denaturation of 94 °C/2 min. followed by 30 cycles of 98 °C/10 sec., 60 °C/30 sec., and 68 °C/30 sec. PCR reactions were performed using the thermal cycler GeneAmp PCR System 9700 (Applied Biosystems/Life Technologies/Thermo Fisher Scientific, Foster City, CA). After PCR amplification, PCR products were directly sequenced using the ABI3130 genetic analyzer (Applied Biosystems/Life Technologies/Thermo Fisher Scientific) in accordance with BigDye terminator method or the fluorescent Dye Terminator Cycle Sequencing method protocol.

### IL37 cDNA characterization

Total RNA was extracted from peripheral blood mononuclear cells (PBMCs) by means of an RNeasy Mini Kit (Qiagen, Courtaboeuf, France). A cDNA fragment of 693 base pairs (bp) was obtained using the one-step reverse transcriptase (RT)-PCR Kit produced by Qiagen (Qiagen, Courtaboeuf, France), with primers (IL37-cDNA-F1: 5′-GTCCTTTGTGGGGGAGAACT-3′; IL37-cDNA-R1: 5′-GGGGCAGTTTCCTAATCGCT-3′). The cycling parameters were as follows: i) 30 min at 50 °C to obtain cDNA (reverse transcription reaction and denaturation of the cDNA template); ii) 15 min at 95 °C (activation of Hot Star Taq polymerase and reverse transcriptase inactivation); and iii) 35 cycles of 30 s at 94 °C for the denaturation step, 30 s at 60 °C for the annealing step, 1 min at 72 °C for the extension step, and 10 min at 72 °C (for the final extension step). When compared to the coding region deduced from the whole genome, the resulting amplified fragments lack nucleotides 26 and 9 at the beginning and end, respectively. The resulting amplified products were separated with agarose gels, purified using QIAquick Gel Extraction Kit (Qiagen, Courtaboeuf, France), and directly sequenced on both strands with the fluorescent Dye Terminator Cycle Sequencing method on a CEQ. 8000 automated sequencer (Beckman Coulter, Villepinte, France). Primers used for sequencing were the same as those used for RT-PCR or, alternatively, two longer primers (IL37-cDNA-F1bis: 5′-GTCCTTTGTGGGGGAGAACTCAGGAG-3; IL37-cDNA-R1bis: 5′-GGGGCAGTTTCCTAATCGCTGACC-3), as well as two additional primers (CLO_IL37_Int_F: 5′-CCTCATCCTTGAGCTCAGCCTC-3; CLO_IL37_Int_R: 5′-GAGGCTGAGCTCAAGGATGAGG-3).

### Expression level of IL37 in macaques infected by SIV

Raw microarray expression data publicly available on the ArrayExpress database (ID: E-MTAB-6068) were used to longitudinally explore IL37 expression in six SIV-infected macaques (for details see Echebli *et al*.^18^). The methods used to generate these data, the ethics statement and meta-information on this experimental study can be found in Echebli *et al*.^18^. Briefly, six cynomolgus macaques exposed intravenously to 5,000 AID50 SIVmac251. The peripheral blood mononuclear cells (PBMCs), peripheral lymph nodes (PLN), and rectal mucosa biopsies (RMB) were collected before inoculation and at both acute (day 9 post-infection: D9) and chronic (month 3 post-infection: M3) phases of infection. Total RNA was isolated from all samples by using the RNeasy RNA isolation kit (Qiagen, CA) and the cDNA microarray profiling was performed as previously described^18^. Briefly, aliquots of 600 ng of labeled RNA were hybridized to Agilent rhesus macaque *Macaca Mulatta* custom 8*60 K array (AMADID 045743, Agilent technologies)^18^. Images of array were analyzed by means of Agilent Feature Extraction software (version 9.5.3.1) following the GE1_1100_Jul11 extraction protocol^18^. All array images passed Agilent QC flags. For each RNA sample, IL37 expression levels were expressed relative to GAPDH levels. The expression fold-changes of D9 and M3 post-infection relative to baseline were used to identify a correlation with the SP-PVL (PVL around day 90 post inoculation). The significance of correlations were assessed by using the Pearson’s and Spearman’s tests.

### Human SNP genotyping

We selected four frequent non-synonymous SNPs located on the human IL37 gene (rs3811047; rs2723192; rs2708947; rs3811046) in order to explore the common IL37 gene structural variation (see Supplementary Table S5 for details). The genotyping was performed by SNP TaqMan Genotyping assays predesigned by Thermo Fisher. Experiments were performed with reagents and protocols provided by Thermo Fisher. The four SNPs were studied in HIV-infected individuals from the French ANRS CODEX cohort of HIV controllers. Patients enrolled in the Codex cohort have to have been infected with HIV-1 for more than five years, have five consecutive plasma HIV RNA viral loads below 400 RNA copies/mL, and must have never received antiretroviral treatment^19^. Each participant in the ANRS HIV Controller CO18/CO21 CODEX cohort provided written informed consent to participate in the study. The study’s objectives and procedures were approved by the local investigational review board (Comité de Protection des Personnes Ile-de-France VII, Paris, France), and the study itself was performed in compliance with the tenets of the Declaration of Helsinki^20^.

## Results

### SNP characterization

High-quality sequence reads obtained from sequencing DNA resulting from exon capture were obtained from six animals (Supplementary Table S3). A total of 19,045 SNPs (ranging from 2,661 SNPs to 3,500 SNPs per animal) were detected by SNP calling, and of these, 13,148 SNPs (from 1,635 SNPs to 2,531 SNPs per animal) were non-synonymous (Table 1). We state that the data mentioned in the article is available upon request. By comparing SP-PVL data generated from the six animals, 12 NS-SNPs located on nine genes were associated with the PVL values. These 12 SN-SNPs were studied in 17 additional animals (23 animals in total) by means of PCR-direct Sanger sequencing, the presence of the SNPs was confirmed, and three SNPs in the IL37, CD244, and IRF9 genes demonstrated high odds ratios (20.05, 9.80, and 4.07 respectively) (Table 2). SNPs of the IL37, CD244, and IRF9 genes were studied in 21 additional animals. The genotyping of these three SNPs in a total of 44 animals revealed high odds ratios and significant association for CD244 (odds ratio: 4.59, *p* = 0.016) and IL37 (odds ratio = 19.52; *p* = 3.3 × 10^−4^). In contrast, the IRF9 SNP showed no significant association (Table 3).

**Table 1:** SNP information.

Sample ID	Total SNP number	SNP number of coding region		SNP number of non-coding region
		Non-synonymous	Synonymous	
9204	3.500	447	522	2.531
11137	2.661	476	550	1.635
11245	3.374	440	546	2.388
Z857	2.854	426	495	1.933
10435	3.222	421	486	2.315
10465	3.434	523	565	2.346
Total	19.045	2.733	3.164	13.148

**Table 2:** Genotypes of 12 NS- SNPs using 14 animals with low PVL (log-PVL <2.7) and 9 animals with high PVL (log-PVL >4.3).

Locus	CD244	IFNGR1	LEF1	IRF9	IRF9	IFIT3	CXCR2	EIF2AK3	IL18RAP	IL37	RANBP2	DDX53
Chromosome	Chr.1	Chr.4	Chr.5	Chr.7	Chr.7	Chr.9	Chr.12	Chr.13	Chr.13	Chr.13	Chr.13	Chr.X
Location	90,806,827	41,537,040	107,555,612	87,945,244	87,945,249	87,895,310	107,701,421	19,755,581	6,821,318	17,064,452	13,295,674	20,943,589
Ref./Var.	T/C	C/T	T/A	A/G	C/T	C/T	G/A	T/C	A/C	C/T	C/T	G/C
Odds ratio	**9.80**	1.08	1.16	**4.07**	**4.07**	2.92	1.04	1.32	1.96	**20.05**	1.64	1.24
95% Cl	1.59 to 110.40	0,28 to 4,21	0.23 to 6.47	1.02 to 18.16	1.02 to 18.16	0.72 to 13.50	0.18 to 5.37	0.34 to 5.14	0.50 to 8.36	2.24 to 986.65	0.31 to 9.79	0.28 to 5.32
*p*	7.62 × 10^−03^	1	1	3.40 × 10^−02^	3.40 × 10^−02^	0.129	1	0.764	0.364	**1.16 × 10** ^**−03**^	0.717	0.753
Animals (route)[log-PVL]												
23037 (IR) [0.000]	C/C	C/T	T/A	A/A	T/T	T/T	G/G	T/C	A/C	C/C	C/T	G/G
14468 (IR) [1.732]	C/C	T/T	T/T	A/A	T/T	C/T	G/G	T/C	A/C	C/C	C/T	C/C
9204 (IV) [1.778]	C/C	C/C	T/A	A/A	T/T	C/T	G/A	T/C	A/A	C/C	C/C	G/G
11137 (IV) [1.778]	C/C	C/C	T/A	A/A	T/T	C/T	G/A	T/T	A/A	C/C	C/C	G/G
11245 (IV) [1.778]	C/C	C/C	T/A	A/A	T/T	C/T	G/A	T/T	A/A	C/C	C/C	G/G
11637 (IV) [1.778]	C/T	T/T	A/A	G/G	C/C	C/C	G/G	C/C	A/C	C/C	T/T	G/G
15885 (IR) [1.959]	C/T	C/T	T/T	A/A	T/T	T/T	G/G	T/C	A/C	C/C	nd	C/C
OBHJ6 (IR) [2.153]	C/C	C/C	T/T	A/A	T/T	T/T	G/G	C/C	C/C	C/C	C/C	G/G
9413 (IV) [2.222]	C/C	C/T	T/T	G/G	C/C	T/T	G/A	T/T	C/C	C/T	C/C	C/C
OBPR6 (IR) [2.283]	C/C	C/T	T/T	A/A	T/T	C/T	G/G	T/C	A/C	C/C	nd	G/G
Z776 (IV) [2.477]	C/C	T/T	T/T	G/G	C/C	C/C	G/G	T/T	C/C	C/C	nd	G/G
10515 (IV) [2.526]	C/C	C/T	T/T	A/G	T/C	C/C	G/G	T/T	A/C	C/C	T/T	C/C
10228 (IV) [2.565]	C/C	T/T	T/T	G/G	C/C	C/T	G/A	T/C	A/C	C/C	nd	G/G
15461 (IR) [2.613]	C/C	C/C	T/A	A/A	T/T	C/T	G/A	C/C	A/C	C/C	T/T	G/G
4763 (IV) [4.326]	C/C	C/T	T/A	G/G	C/C	C/T	G/G	T/T	A/C	C/C	C/C	G/G
8102 (IV) [4.506]	C/C	C/T	T/T	G/G	C/C	C/T	G/G	T/T	A/A	T/T	C/C	G/G
20483 (IV) [4.586]	C/C	C/T	T/A	A/G	T/C	T/T	G/A	T/T	A/C	C/T	nd	G/G
11360 (IV) [4.828]	T/T	C/C	T/T	G/G	C/C	C/T	G/A	C/C	A/A	C/C	C/C	G/G
Z860 (IV) [4.968]	C/T	C/C	T/A	A/A	T/T	C/C	G/A	C/C	A/A	T/T	C/T	G/G
Z857 (IV) [5.239]	C/T	C/T	T/T	G/G	C/C	C/C	G/G	T/C	A/C	C/T	C/C	C/C
OBRF6 (IR) [5.265]	T/T	C/T	T/A	A/A	T/T	C/C	G/A	C/C	A/C	C/C	nd	G/G
10435 (IV) [5.399]	C/T	T/T	T/T	G/G	C/C	C/C	G/G	T/C	A/C	C/T	T/T	C/C
10465 (IV) [5.559]	C/T	C/T	T/T	A/G	T/C	C/C	G/G	T/C	A/C	C/T	C/T	C/C

The odds ratio and 95% confidence interval (CI) were calculated as detailed in Materials and Methods.

Probabilities of association (*p*) were obtained using the Fisher exact test. Boldface **p** values indicate significance levels <5 × 10^−2^. Applying the Bonferroni correction (because we studied 11 candidate genes, the threshold of significance is 4.5 × 10^−3^), only IL37 was had a significant association with PVL. In the case of IRF9, because the two SNPs yielded homologues genotypes in 23 animals, only one of the two SNPs was studied for the additional animals (see Table 3).

**Table 3:** Genotypes of the three NS-SNPs with 22 animals above the median log-PVL and 22 animals below the median log-PVL.

Locus name	CD244	IRF9	IL37 (IL37)
Chromosome	Chr.1	Chr.7	Chr.13
Location	90,806,827	87,945,244	17,064,452
Ref./Var.	C/T	A/G	C/T
Odds Ratio	4.59	1.34	**19.52**
95% confidence	1.27 to 21.09	0.51 to 3.57	2.70 to 863.87
*p*	0.016	0.656	**3.33 × 10** ^**−4**^
**Animals (route) [log-PVL]**			
23037 (IR) [0.000]	C/C	A/A	C/C
14468 (IR) [1.732]	C/C	A/A	C/C
9204 (IV) [1.778]	C/C	A/A	C/C
11137 (IV) [1.778]	C/C	A/A	C/C
11245 (IV) [1.778]	C/C	A/A	C/C
11637 (IV) [1.778]	C/T	G/G	C/C
15885 (IR) [1.959]	C/T	A/A	C/C
OBHJ6 (IR) [2.153]	C/C	A/A	C/C
9413 (IV) [2.222]	C/C	G/G	C/T
OBG7 (IR) [2.228]	C/C	A/A	C/C
OBPR6 (IR) [2.283]	C/C	A/A	C/C
Z776 (IV) [2.477]	C/C	G/G	C/C
10515 (IV) [2.526]	C/C	A/G	C/C
10228 (IV) [2.565]	C/C	G/G	C/C
15461 (IR) [2.613]	C/C	A/A	C/C
11296 (IV) [2.771]	C/C	A/G	C/C
OBFE6 (IR) [2.899]	C/C	A/G	C/C
9691 (IV) [2.905]	C/C	A/A	C/C
20595 (IV) [2.964]	C/C	A/A	C/C
8249 (IV) [2.998]	C/C	A/A	C/C
23060 (IR) [3.094]	C/T	A/G	C/C
20351 (IR) [3.094]	C/T	G/G	C/C
20654 (IV) [3.137]	C/C	A/A	C/C
15232 (IR) [3.273]	T/T	A/G	C/T
10116 (IV) [3.398]	C/C	A/A	C/C
15693 (IV) [3.428]	C/C	A/A	C/T
473 (IV) [3.499]	T/T	A/G	C/C
10024 (IV) [3.544]	C/T	A/A	C/C
9345 (IV) [3.614]	C/C	A/A	C/C
20525 (IV) [3.925]	C/C	A/G	C/T
9859 (IV) [4.004]	C/C	A/A	C/T
8141 (IV) [4.053]	C/C	A/G	C/C
15596 (IV) [4.053]	C/T	A/A	C/T
23014 (IR) [4.124]	C/C	A/A	C/T
OBRG6 (IR) [4.262]	C/C	A/G	C/C
4763 (IV) [4.326]	C/C	G/G	C/C
8102 (IV) [4.506]	C/C	G/G	T/T
20483 (IV) [4.586]	C/C	A/G	C/T
11360 (IV) [4.828]	T/T	G/G	C/C
Z860 (IV) [4.968]	C/T	A/A	T/T
Z857 (IV) [5.239]	C/T	G/G	C/T
OBRF6 (IR) [5.265]	T/T	A/A	C/C
10435 (IV) [5.399]	C/T	G/G	C/T
10465 (IV) [5.559]	C/T	A/G	C/T

The odds ratio and 95% confidence interval (CI) were calculated as detailed in Materials and Methods. Probabilities of association (*p*) were obtained using the Fisher exact test. Significant *p* values are represented in bold.

We tested the effect of these three gene variants on the PVL using a linear model. We also introduced information on the MHC haplotypes, since these haplotypes were previously shown to influence PVL. The seven haplotypes described in this population were treated as binary variables (presence or absence). Starting from the full model, we removed the non-significant terms in a stepwise manner. This left only three significant predictors, namely IL37 SNP (*p* = 0.0026), M2 (*p* = 0.0027), and M6 (*p* = 0.0023) haplotypes. These three factors in conjunction explained 48% of the PVL variance (*p* = 4.8 × 10^−6^).

### Multiple linear regression model

Since it has been demonstrated that the MHC class IB region influences the control of SIV replication at the set point, we decided to study the combined influence of the MHC genotype and the three NS-SNPs (CD244; IL37; IRF9) on the SP-PVL.

The combined effect of the three gene variants (CD244; IL37; IRF9) and seven MHC class IB haplotypes on the log SP-PVL was tested by using a multivariate linear model. The SNP variants and MHC haplotypes were treated as binary variables (presence or absence) as described in materials and methods. The log SP-PVL was treated as a quantitative trait. Forward selection or backward elimination procedures led to the same best model encompassing three significant predictors, including the IL37 NS-SNP (Chr13:17,064,452) and the M2 and M6 MHC class IB haplotypes. The combination of these three factors explained 48% of the PVL variance (p = 4.8 × 10^−6^). Using ANOVA, association of the three factors was highly significant (IL37 SNP (p = 0.0026); M2 (p = 0.0027); M6 (p = 0.0023)). Alternative models were tested by incorporating NS-SNPs characterized in our previous study^15^ (see Supplementary Table S6 and discussion). In all of the models we tested, the IL37 NS-SNP was the one with the most significant association with the PVL. In total, one NS-SNP located in exon 1 of the IL37 gene (chr.13: 17,064,452) was significantly associated with the SP-PVL. Most cynomolgus macaque alleles (73/88 alleles) have a “C” in this position (corresponding to a threonine, ACG codon), while the minor allele is a “T” (corresponding to a methionine, AUG codon as in humans).

### Study of the macaque IL37 gene exon 1

**Figure 1:**
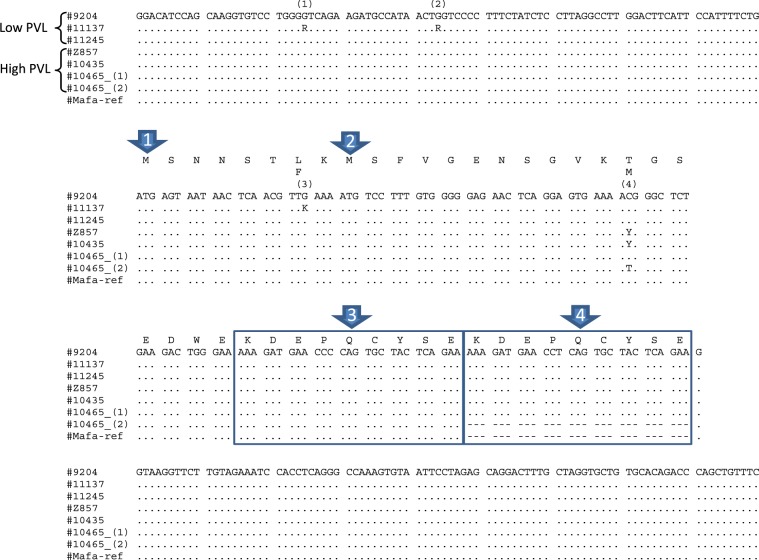
The polymorphic positions (1), (2), (3) and (4) correspond to positions 17064328, 17064347, 17064414, and 17064452 of Mafa chr.13 (whole genome annotation v5.0), respectively. The SNP at position (4) (Chr.13 :17064452) was significantly associated to the SP-PVL. Mafa-ref correspond to the sequence of the Macaca_fascicularis_5.0 genome annotation. Arrow 1. The macaque exon 1 displays an in-frame ATG codon nine codon upstream of the ATG start codon of the human IL-37 gene. In the human genome, this start codon is absent. Arrow 2. Location of the human IL-37 start codon. Arrow 3. A 27 bp motif is duplicated in the most frequent IL-37 macaque alleles studied here. This insertion was absent in one allele of animal 10465_IL1F7. A similar allele was found in another animal (animal 8102) among the 44 Mauritian cynomolgus macaques studied here (see text for details). Arrow 4. The 27 bp repeat is absent from Mafa and Mamu whole genome annotations. It is also absent from the human IL-37 gene. The 3′ duplicated 27 bp motif differs from its homologue by one base (there is a T at position 207 instead of a C at position 180). This substitution is synonymous.

We characterized the IL37 exon 1 sequence (531 bp) in 44 animals by PCR and direct sequencing. The alignment of the most frequent sequences is presented in Fig. 1. The variants outside exon 1 are described in Supplementary Table S7. By comparing it to human IL37 exon 1, the cynomolgus IL37 exon 1 displays i) an in-frame ATG codon 24 bp upstream of the human ATG start codon, and ii) a duplication of a 27 bp motif. In only two of the 44 animals, we observed the presence of alleles missing the duplication of the 27 bp motif (animals #8141 and #10465). The variation of repeat number was confirmed by a nested PCR of exon-1 and by sequencing the shortest amplified fragment.

### Characterization of cynomolgus macaque IL37 coding sequences

**Figure 2:**
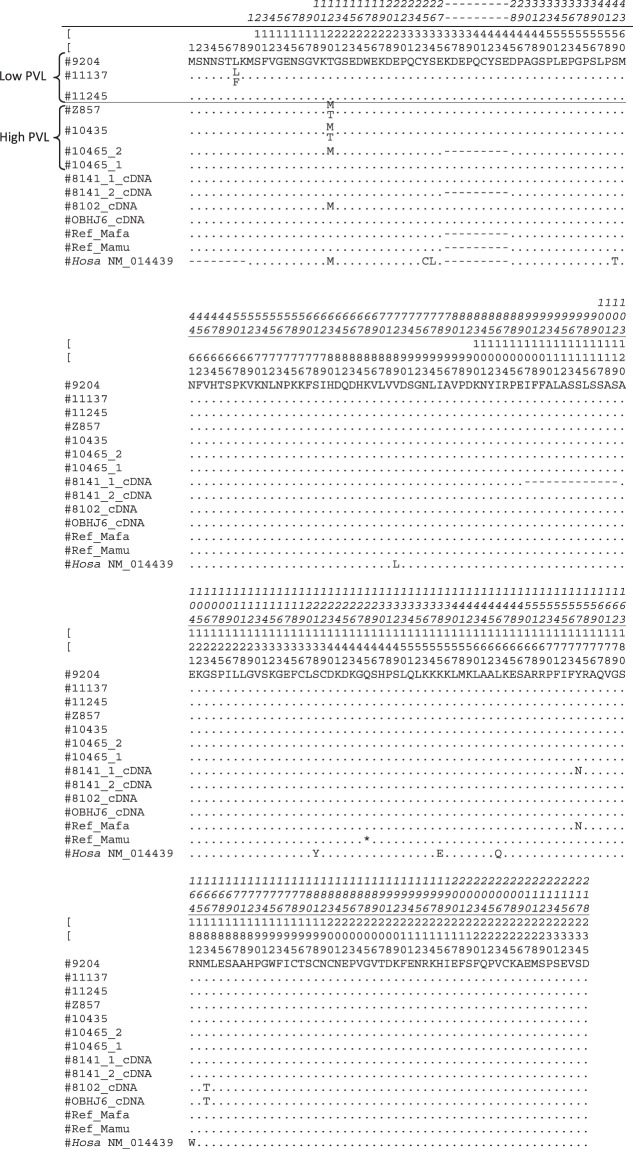
The amino acid positions are numbered by reference to the macaque sequences characterized in this study (number in italics) or by reference to the human IL37 isoform b (NP_055254 corresponding to coding sequence #NM014439). The macaque protein sequences were deduced from cDNA sequences of animals #8141, #8102 and #OBHJ6 that were characterized from cDNA amplified by RT-PCR from buffy coats RNA (see Materials and Methods for details). The 46 first bases in 5′ were characterized at the genomic level by amplifying and sequencing exon 1 and the surrounding regions. Other macaque protein sequences were deduced from genomic sequences of six animals (#9204, #11137, #11245, #Z857, #10435 and #10465), which were characterized in these six animals by means of sequence capture. The NS variant associated with PVL is responsible for the amino acid polymorphism (T- > M) at position 20. Animals #Z857 and #10435 are heterozygous at this position. Animal #11137 was heterozygous for a non-synonymous variant (codon 7) that is not associated with PVL. Two animals (#10465 and #8141) have alleles (“#10465_2” and “#8141_2”) missing the 27 bp repeat in exon 1 (for more details, see text). Ref_Mafa and Ref_Mamu sequences correspond to XP_015288882 (predicted transcript XM_015433396) and XP_002808097 (predicted transcript XM_002808051), respectively. Note that the *Mamu* IL-37 predicted mRNA sequence (XM_002808051) present a nonsense mutation at codon 136 (homologous to codon 145 of Mafa) that is indicated by a “*” in the figure. Another *Mamu* IL-37 predicted amino acid sequence (EHH22430; locus tag: EGK_05691) does not have a stop codon at position 136 and is 8 amino acids shorter than XP_002808097 at the amino-terminal extremity^2^. All *Mafa* or *Mamu* IL37 coding sequences available in databanks lack the 27 bp motif duplication in exon1.

The IL37 coding sequences of six animals were characterized at the genomic level. We also characterized the sequence of IL37 transcripts of three animals. The IL37 cDNA amplified from total RNA extracted from peripheral blood mononuclear cells (PBMCs) were sequenced. The macaque cDNA sequences corresponded to the human splicing variant 01 (NM014439 encoding isoform B protein NP_055254). We characterized two alleles in animal 8141 distinguishing between the presence/absence of the 27 bp nucleotide repeat (see Fig. 2 for details). For two other animals (#8102 and #OBHJ6), the repeat was present in the two alleles. The SNPs located within the coding region of the IL37 gene are described in Supplementary Table S8. The SNPs that were characterized in the rhesus monkey IL37 coding sequence were imported from the NCBI databank and are depicted in Supplementary Table 9. It is important to note that the NS-SNP associated to the PVL in the cynomolgus macaque model was also described in rhesus monkey.

**Figure 3:**
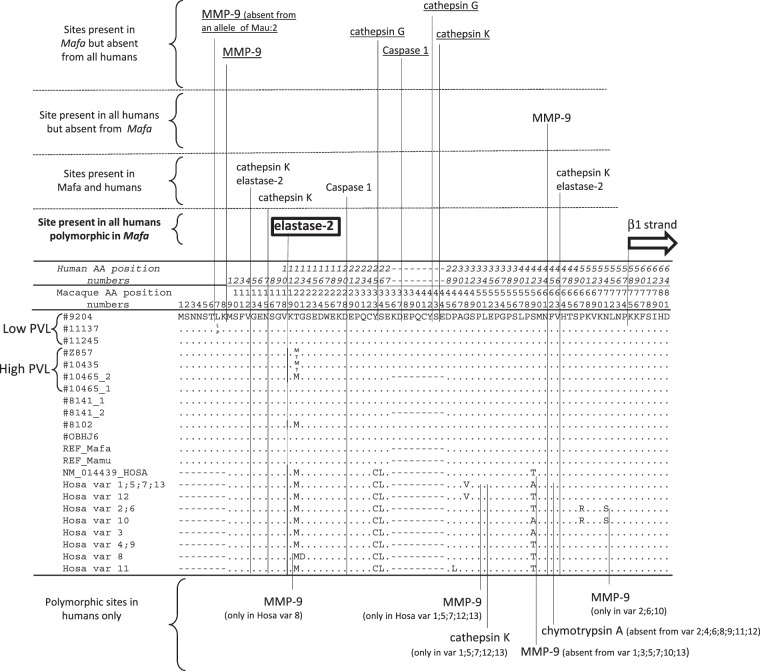
Macaque sequences are described in the legend for Fig. 1. The human variants (var 1 to 13) are from Kang *et al*.^47^. Enzyme cleavage sites were predicted using a Prosper server^55^. The underlined predicted cleavage sites are those observed in macaque proteins but not in human ones. The amino acid substitution observed at position 7 does not modify the MMP9 cleavage site. The presence of a threonine at position 20 of macaque protein (instead of a methionine as in other macaque variants and as in human and most nonhuman primates) destroys an elastase 2 cleavage site (rectangle). Six human polymorphic cleavage sites are absent from macaque proteins. Also note that the macaque proteins can have two caspase 1 cleavage sites instead of only one in human proteins (and rare macaque variants). The second caspase 1 cleavage site (rectangle) of macaque proteins is encoded by a duplicated 27 bp motif.

The protein sequences deduced from macaque IL37 cDNA showed a high degree of conservation with their human counterpart (human isoform B encoded by the human splicing variant 01). As various enzymatic cleavages in the amino-terminal portion of IL37 are required to produce active forms of the protein, we analyzed the potential enzyme cleavage sites in this region (Fig. 3). The caspase-1 cleavage site characterized in the human sequence (“KDE” human positions 19–21) is conserved in the macaque sequence. A second KDE motif is present in the macaque sequence due to the presence of nine additional amino acid motifs (encoded by the duplicated 27 bp motif in exon 1). Interestingly, the number of nine amino acids motifs is variable among different primate species, leading to a variable number of caspase 1 cleavage sites (Supplementary Figs S1 and S2).

### IL37 Expression level in macaque infection by SIV

By studying the relative expression level of IL37 in various tissues (RMB, PLN, PBMC) of the six animals, we found that IL37 expression level on D9 or M3 after inoculation did not significantly change compared to the level before inoculation (D0) (Supplementary Fig. S3). Only on D9 in RMB a slight increase of expression was observed with a significant negative correlation between the SP-PVL and the fold-change of IL-37 expression at D9 in the RMB (R² = 0.89, p = 0.0047 Pearson’s test; r_s_^2^ = 1, p = 0.0027 Spearman’s test) (Supplementary Fig. S4). In contrast, in PBMCs and LN, we observed on D9 and M3 a non-significant decrease of IL37 expression level and fold-changes in IL37 expression in RMB at M3 or in PBMCs and PLNs on D9 and M3 did not correlate with the SP-PVL. All animals were genotyped for the IL37 NS SNP associated with control of SIV infection. One animal was heterozygous for this SNP, while the other five were homozygous. Before and after to inoculation of SIV, the heterozygous animal had among the lowest values of IL37 expression level in all the organs studied with only one exception on D9 in LN.

### Study of IL37 polymorphisms in humans infected with HIV

Since an association between macaque IL37 gene polymorphism and control of SIV infection was observed, we decided to explore the polymorphism of the human IL37 gene in a cohort of HIV-infected patients in whom viral replication is spontaneously undetectable (HIV controllers). As described in the Methods section, four non-synonymous SNPs located on the human IL37 gene were selected and genotyped in 171 individuals (54 Africans and 117 Caucasians) from the ANRS CODEX by means of quantitative PCR (Life Technologies). The allele frequencies were compared to those reported for Africans and Caucasians in the 1000 genomes dataset. We did not observe any significant differences between allele frequencies in the CODEX cohort and reference data for the corresponding populations (Table 4).

**Table 4:** Comparison of IL37 allele frequencies in HIV controllers and the corresponding reference populations (1000 genomes data set).

**rs2708947**					
**Population**	**Observed** ^**a**^		**Deduced** ^**b**^		***p*** ^**c**^
	**C**	**T**	**C**	**T**	
Caucasians	22	212	19	215	0.7441
Africans	18	90	19	89	0.8592
**rs2723192**					
**Population**	**Observed** ^**a**^		**Deduced** ^**b**^		***p*** ^**c**^
	**A**	**G**	**A**	**G**	
Caucasians	21	213	19	215	0.7446
Africans	18	88	18	88	>0.9999
**rs3811046**					
**Population**	**Observed** ^**a**^		**Deduced** ^**b**^		***p*** ^**c**^
	**G**	**T**	**G**	**p** ^**b**^	
Caucasians	81	155	71	165	0.3753
Africans	84	24	89	19	0.4012
**rs3811047**					
**Population**	**Observed** ^**a**^		**Deduced** ^**b**^		***p*** ^**c**^
	**A**	**G**	**A**	**G**	
Caucasians	80	156	71	165	0.3768
Africans	76	30	81	25	0.6230

^a^Allele numbers observed among HIV controllers (CODEX cohort). For some individuals, we did not obtain the genotype of the four SNPs, so the sums of alleles vary from one SNP to another.

^b^The deduced allele numbers were calculated using the allele frequencies from the 1000 genomes dataset.

^c^Fisher’s exact test probabilities.

## Discussion

In the experimental model of SIV infection of macaques originating from Mauritius, we search for associations between the SP-PVL and polymorphisms in the exons of 390 immune-related genes. The initial search was based on the study of six animals, three each of the highest and lowest SP-PVL, respectively. From the initial screening, we retained three candidates that were studied out of a total of 44 animals. Taking into account the Bonferroni correction, only one NS-SNP in exon 1 of the IL37 gene was significantly associated with the SP-PVL. As discussed below, this NS-SNP determines the presence/absence of an elastase site that could affect the maturation of the secreted pro-IL37. As the presence of the elastase site is associated with high SP-PVL values, we hypothesize that the elastase-dependent maturation of the secreted IL37 is probably deleterious in the control of SIV infection in the macaque model. Moreover, using a multivariate model, we found only three significant predictors of SP-PVL, including the IL37 NS-SNP (*p* = 0.0026), and the MHC Class IB haplotypes M2 (*p* = 0.0027) and M6 (*p* = 0.0023). These three factors together explained 48% of the PVL variance (*p* = 4.8 × 10^−6^).

It is important to note that the IL37 NS-SNP (chr.13: 17,064,452) characterized here was not detected in our previous study^15^, in which we used complete genome sequencing of nine animals (three each with the highest, intermediate, and lowest set-point PVL) to detect candidate NS-SNPs. The discrepancy between the two studies is due to the fact the only four animals used in each of the two screenings (two animals each with the highest and lowest PVLs, see Supplementary Table S1). The genotypes of the nine animals chosen for the whole genome sequencing association study did not include the IL37 NS-SNPs among candidate SNPs. In fact, the low numbers of animals used in the two studies introduce a high risk of sampling effect. For example, if one selected three animals at random among those with a log-PVL lower than 2.4, and three animals with a log-PVL above 4.1, the probability of having a sample of six animals allowing the detection of the potential association of the IL37 NS-SNP is only 0.57. With the rules from the whole genome association used for detecting candidates, the probability of having a sample of nine animals allowing the detection of IL37 NS-SNPs was only 0.14. As detailed in our previous article^15^, the low number of animals used for the first detection step of candidate SNPs results in a high probability of discrepancy from one study to another. For the same reason, the IL37 gene was also not detected in the study performed by Ericsen *et al*.^14^, in which 12 animals were used to detect candidate SNPs. On the other hand, it is interesting to note that in the whole genome association study, we detected SNPs in the 3′UTR of the IL36 gamma (IL36G) gene. Interestingly, the IL36G is the closest neighbor gene to IL37 on chromosome 13 of the *Mafa* genome. Since the IL36G gene and non-coding region were not included in the present study, we did not detect the IL36 3′UTR SNP. As shown in Supplementary Table S10, the genotypes of 42 animals were very similar for IL36G and IL37 SNPs. The study of IL36G and IL37 polymorphism on a larger number of animals experimentally infected with SIV is needed in order to define the best candidate for both genes.

Because of their high degree of similarity, the two IL36G SNPs and the IL37 SNP were not compatible in multiple linear regression models. However, other candidate SNPs characterized in our previous study were compatible with IL37 SNP in an MLR model (Supplementary Table S6**)**. In this MLR model, five markers (including M2 and M6 MHC haplotypes, IL37, MAGED4 and CD244) in combination explained 67% of the log-PVL variance (*p* = 7.7 × 10^−9^) (Supplementary Table S6). The potential role of MAGED4 in the control of SIV was discussed in our previous article^15^. The potential role of IL37 NS-SNP is discussed further. As for the CD244 gene, for the Mauritius cynomolgus macaques studied here, we observed two NS-SNPs in exon 9. A “T” instead of a “C” at position Chr1: 90806827 causes a premature stop codon (instead of an arginine), resulting in the loss of 14 amino acids at the carboxylic terminal of the CD244 protein. The (G- > A) polymorphism at position Ch1: 90806848 is linked to the previous one and causes amino acid change E- > K (Supplementary Fig. S5**)**. CD244 (also referred to as 2B4) is a member of the Signaling Lymphocyte Activation Molecule (SLAM) family^21^. It is expressed on NK cells as well as on a subset of memory T cells. Its ligand is CD48, and the CD244/CD48 interaction regulates target cell lysis by NK cells and CD8+ cytotoxic T cells, in addition to participating in the fight against viral infection and regulation of effector/memory T cell generation and survival^22,23^. The receptor CD244 exhibits both inhibitory and activating functions^24^. Human CD244 has four immune tyrosine-based switch motifs (ITSM) in its cytoplasmic tail. These motifs have been shown to interact with numerous small adaptor molecules through SH2 domains^25^. Association between CD244 and SAP is independent of phosphorylation of ITSMs, while binding of all other adaptor proteins is phosphor-dependent. The adaptor SAP and EAT-2 activate the functions of NK cells, while other adaptor proteins such as phosphatases SHP-1, SHP-2, and SHIP may be responsible inhibiting NK functions^26^. Sustained high-level expression of 2B4 on virus-specific CD8+ T cells is characteristic of persistent viral infections^27^. For example, 2B4 is upregulated on CD8+ T cells collected from patients with persistent HIV infection^28^. It can be deduced that the loss of 14 amino acids at the carboxylic terminal of the CD244 protein leads to impaired CD244-dependent inhibition of NK cells, which could lyse SIV-specific effector memory T CD8+ cells (based on similarities to observations in the LCMV mouse model)^29^. Alternatively, one could hypothesize that the shortened CD244 protein would not allow sufficient co-activation of SIV-specific effector memory T CD8+ cells or would promote strong inhibition of these cells. In both cases, it would be important to check all these hypotheses by comparing the functionality of SIV-specific T cells in animals having a C/C or a T/T genotype at position Chr1: 90806827.

Because the probability of association of PVL was highest with IL37 NS-SNP (chr.13: 17,064,452) (Table 3), we will focus our final discussion on the IL37 gene. IL37 is a very unique cytokine with an anti-inflammatory effect^30–32^. It belongs to the IL-1 family and interferes with many cytokines that induce inflammation, such as IL-1A, IL-1B, IL-18, and IL-33^33–37^. The human IL37 gene is located on chromosome 2q12–13 in the middle of a chromosomal region containing many gene-encoding cytokines, including the IL1-A and IL1-B genes^38^. IL37 has two modalities of action. The first is dependent upon the secretion of the IL37 gene, which is cleaved by unknown extracellular enzymes and binds to its receptor, which then encompasses two membrane proteins, respectively: the beta chain of the IL-18 receptor (IL-18RB) and the orphan IL1-R8 protein, the latter of which is responsible for the inhibitory signal^39^. The second pathway requires the cleavage of intracytoplasmic IL37 by caspase-1. The caspase 1-cleaved IL37 translocates into the nucleus, where it combines with Smad3 to modulate the expression of several genes involved in inflammation^30,40^. In particular, the IL37-Smad3 complex induces decreased transcription of IL-6 and IL-1B^41^. Since the IL37 gene is absent in mice, many experiments are based on transgenic mouse into which the human IL37 gene has been introduced, as reviewed by Dinarello *et al*.^37^. These knock-in mice are resistant to concavalin A-induced hepatitis^42^, LPS toxic shock^43^, and dextran sulfate induced colitis^44^. Other experimental data proved that IL37 is capable of impairing adaptive immune responses by inducing tolerogenic dendritic cells^45^ and favoring induction of Treg cells^46^. The human IL37 gene has many polymorphisms and recently, it was demonstrated that two major haplo-groups are present in human populations^47^. A recent study demonstrated that the human variants possess functional differences due variation in their destruction rates^48^. Previous reports suggested that IL37 was involved in the development of various inflammatory conditions, including ankylosing spondylitis and rheumatoid arthritis^49–51^. More recently, expression of the IL37 gene was shown to be increased in individuals with chronic HIV-1 infection^52^. It has been demonstrated that when human IL37 is secreted, the most active form was cleaved between phenylalanine (position 45) and valine (position 46 of the human IL37b) by an unknown protease^53^. Human IL37b with the N-terminus at valine 46 was found to be produced naturally in the supernatant of a eukaryote-transfected cell line^54^. Therefore, the position of the amino acid change related to *Mafa* NS-SNP (Chr.13:17064452) is located outside the active forms of the protein. However, this does not preclude that the amino acid variant could affect the maturation of pro-IL37. We analyzed the potential cleavage sites in the macaque proteins by means of the PROSPER server^55^. As shown in Fig. 3, the IL37 *Mafa* NS-SNP (Chr.13:17064452) is responsible for a threonine/methionine amino acid polymorphism at position 20 of the macaque protein (homologous to position 12 of the human protein). In humans (all known variants) and in all non-human primates (except cynomolgus and rhesus macaques), the homologous amino acid position is occupied by a methionine. The presence of a threonine in the macaque protein destroys an elastase 2 cleavage site. In Mauritian macaques, the presence of a methionine was associated with high PVL. Therefore, the presence of the elastase site in macaque pro-IL37 is associated with high PVL in the SIV macaque model. If this elastase site favors the maturation of the secreted pro-IL37, then easier maturation would be associated with high PVL. The alternative hypothesis is that the NS-SNP (*Mafa* Chr.13:17064452) is linked to another uncharacterized variant in the region that controls the expression of IL37, or nearby gene. As indicated above, in a previous study we characterized one SNP in the 3′UTR region of the IL36G gene^15^. At this stage, it is not yet possible to conclusively select the best candidate gene in the IL37/IL36G region, because of the insufficient number of genotyped animals.

At this stage of the discussion, it is important to recall that the IL37 gene is absent from the chimpanzee genome^56^. The deletion of the IL37 gene is observed in all members of all chimpanzee subspecies that have been studied thus far, meaning that deletion of the IL37 gene can been considered fixed in the common ancestor of all living chimpanzee species^57^. In contrast, the IL37 gene is present in the genome of other anthropoid apes (gorillas and orang-utan) and monkeys (Supplementary Figs S1 and S2). Therefore, chimpanzees and mice are the only known mammals that have lost the IL37 gene. The loss of IL37 in chimpanzees’ ancestors has no evident pathological consequences. Indeed, chimpanzees are, by and large, as resistant as humans to various infectious diseases, and chimpanzees do not suffer from uncontrolled inflammatory diseases. The IL37 gene appears dispensable and its absence in chimpanzees is most probably compensated by another cytokine. Since SIVcpz epidemia did not start in the common ancestor species of living chimpanzee species^58,59^, it can be reasonably inferred that SIVcpz epidemia did not positively select IL37 gene deletion in chimpanzees. Thus, the absence of the IL37 gene in chimpanzees is likely not related to their better resistance (compared to humans) to developing lentivirus-related AIDS. However, if one presumes that it is related, then the expression of IL37 should be deleterious in humans and macaques. Moreover, although Chattergoon *et al*.^60^ demonstrated that IL37 inhibits HIV replication in CD4+ human lymphocytes, it is important to note that chimpanzees, who have lost the IL37 gene, control lentivirus infections (SIVcpz in the wild and HIV in experimental conditions) far better than humans do. Therefore, the direct effect of IL37 on lentivirus replication reported by Chatergoon *et al*. is most probably not determinant in the control of lentivirus infection. In addition, IL37 was reported to suppress antigen-specific adaptive immunity via the induction of tolerogenic dendritic cells^45^, and to inhibit T-cell priming by modulating dendritic cell maturation^61^.

In order to shed some light on the role of IL37 in the control of the SIV infection, we quantified the expression of IL37 in various tissues of macaque infected by SIV. In the macaque model, there is no significant change in the level of IL37 expression after SIV inoculation. However, nine days after inoculation, the fold-change in IL37 expression in the RMB was negatively correlated with the SP-PVL (Supplementary Fig. S3). This could suggest that an increase in IL37 expression contributes to the control of SIV replication in the digestive tract at the acute phase of the infection, favoring a better control of the infection at the set point. In lymph node on D9, and in PBMCs on M3 we observed a decrease of IL37 expression (Supplementary Fig. S4). However, the change of IL37 expression level in PBMCs or PLN at D9 or M3 after inoculation was not correlated with the SP-PVL, suggesting that the level of expression of IL37 in blood and lymph nodes is not associated with control of SIV infection. It is important to note that contrary to what is observed in patients infected by HIV^52^, IL37 expression level does not increase in PBMCs of macaques infected by SIV. In total, IL37 could be beneficial in the control of SIV infection by decreasing the inflammation associated with a strong virus replication in the acute phase of the infection. Alternatively, IL37 could be detrimental to the SIV infection control by inhibiting the presentation of SIV peptides by dendritic cells. In the latter hypothesis, IL37 would impair the strength of the cellular immune response that plays a crucial role in the control of SIV infection. In order to separate these two hypotheses, the injection of recombinant IL37 or IL37-blocking antibodies into SIV-infected macaques would be necessary.

It is important to note that the IL37 NS-SNP that we have characterized in Mauritian macaques does not exist in humans. However, we explored a possible association between polymorphism of human IL37 and control of HIV infection. Among four NS-SNPs of the IL37 gene in a cohort of HIV controllers (CODEX cohort), we defined the main IL37 alleles observed in European and African populations^47^. Based on their data, Kang *et al*.^47^ recommended genotyping any one of the five non-synonymous SNPs (rs2708943: C > G, rs2723183: A > G, rs2723187: C > T, rs2708947: C > T, or rs2723192: A > G) to differentiate between IL37 haplogroups 1 and 2, which differ at least at five non-synonymous sites and could result in differing susceptibility to human diseases. We used two of the five recommended SNPs (rs2708947:C4T and rs2723192:A4G) to differentiate between the two main IL37 haplogroups. We did not find evidence of any significant difference in allele frequencies after comparing it to the general reference population.

In total, an NS-SNP IL37 is significantly associated with the control of SIV infection in Mauritian cynomolgus macaques. However, further studies are needed to define how IL37 interferes with the control of SIV infection. Since control of SIV infection *in vivo* is a complex process involving a combination of innate and adaptive immunity, *in vitro* reductionist models may not be fully suitable for experimentally demonstrating the effect of IL37 on control of SIV infection. *In vivo* experiments (by injecting either blocking antibodies or recombinant macaque IL37) might be more appropriate in this endeavor.

## Supplementary information

Supplementary Tables and Figures
