# Supplementary material for: Cynomolgus macaque IL37 polymorphism and control of SIV infection
Source: Sci Rep. 2019 May 28;9:7981. doi: 10.1038/s41598-019-44235-x (PMC6538695; doi:10.1038/s41598-019-44235-x)
Supplement: Supplementary file 1 — Supplementary Tables and Figures [file 41598_2019_44235_MOESM1_ESM.docx]

**Title: Cynomolgus macaque IL37 polymorphism and control of SIV infection**

**Running title: IL37 polymorphism and control of SIV infection**

Takashi Shiina^1^, Shingo Suzuki^1^, Nicolas Congy-Jolivet^2,3^, Alice Aarnink^2^, Henri-Jean Garchon^4^, Nathalie Dereuddre-Bosquet^5^, Bruno Vaslin^5^, Nicolas Tchitchek^5^, Delphine Desjardins^5^, Brigitte Autran^6^, Olivier Lambotte^5,7^, Ioannis Theodorou^8^, Roger Le Grand^5^, Antoine Blancher^2,3,9^

^1^ Department of Molecular Life Sciences, Division of Basic Medical Science and Molecular Medicine, Tokai University School of Medicine, 143 Shimokasuya, Isehara, Kanagawa 259-1193, Japan

^2^ Laboratoire d’immunogénétique moléculaire (LIMT, EA 3034, Faculté de médecine Purpan, Université Toulouse 3, Paul Sabatier, UPS)

^3^ Laboratoire d’immunologie, CHU de Toulouse, Institut Fédératif de Biologie, hôpital Purpan, 330 Avenue de Grande Bretagne, TSA40031, 31059 Toulouse cedex 9, France.

^4^ Inserm U1173, Simone Veil School of Health Sciences, University of Versailles Saint-Quentin-en-Yvelines, Montigny-le-Bretonneux, France; Genetics Division, Ambroise Paré Hospital (AP-HP), Boulogne-Billancourt, France

^5^ CEA – Université Paris-Sud 11 – INSERM U1184, Immunology of Viral Infections and Autoimmune Diseases, IDMIT Department, IBFJ, 92265, Fontenay-aux-Roses, France

^6^ U1135, CIMI, INSERM, Paris, France, Sorbonne Universités, UPMC Université Paris 06, Paris, France, Département d'Immunologie, Hôpital Pitié-Salpêtrière, AP-HP, Paris, France

^7^ Assistance Publique – Hôpitaux de Paris, Service de Médecine Interne et Immunologie Clinique, Groupe Hospitalier Universitaire Paris Sud, Hôpital Bicêtre, Le Kremlin‐Bicêtre, France, Université Paris Sud, Le Kremlin Bicêtre, France,

^8^ Center for Immunology and Infectious Diseases, INSERM UMR S 1135, Pierre et Marie Curie University, Paris, France,

^9^ Centre de Physiopathologie Toulouse-Purpan (CPTP), Université de Toulouse, Centre National de la Recherche Scientifique (CNRS), Institut National de la Santé et de la Recherche Médicale (Inserm), Université Paul Sabatier (UPS), Toulouse, France.

**Corresponding author E mail address**: [blancher.antoine@neuf.fr](mailto:blancher.antoine@neuf.fr)

**Supplementary Tables and Figures.**

**Supplementary Table S1: Animals included in this study (N=44).**

| Animal ID# | Animal identity code ^(1)^ | Route of inoculation ^(2)^ | Log PVL value at the set point ^(3)^ | MHC haplotype in the class IB region ^(4)^ |
| --- | --- | --- | --- | --- |
| 1 | 23037 | IR | 0.00 | M2/M6 |
| 2 | 14468^(6)^ | IR | 1.73 | M1/M4 |
| 3 | **#9204** WGS^(5^**^)^** | IV | 1.78 | M4/M6 |
| 4 | **#11137** | IV | 1.78 | M2/M5 |
| 5 | **#11245** WGS^(5)^ | IV | 1.78 | M3/M2 |
| 6 | 11637 | IV | 1.78 | M3/M2 |
| 7 | 15885 | IR | 1.96 | M7/M2 |
| 8 | OBHJ6 | IR | 2.15 | M3/M6 |
| 9 | 9413 WGS^(5)^ | IV | 2.22 | M3/M1 |
| 10 | OBG7 | IR | 2.23 | M2/M6 |
| 11 | OBPR6 | IR | 2.28 | M2/M4 |
| 12 | Z776 | IV | 2.48 | M5/M6 |
| 13 | 10515 | IV | 2.53 | M2/rec ^(4)^ |
| 14 | 10228 | IV | 2.57 | M3/rec*** |
| 15 | 15461 | IR | 2.61 | M2/M3 |
| 16 | 11296 | IV | 2.77 | M3/M2 |
| 17 | OBFE6 | IR | 2.90 | M1/M2 |
| 18 | 9691 | IV | 2.91 | M3/M3 |
| 19 | 20595 | IV | 2.96 | M2/rec ^(4)^ |
| 20 | 8249 WGS^(5)^ | IV | 3.00 | M1/M4 |
| 21 | 23060 | IR | 3.09 | M3/M3 |
| 22 | 20351 | IR | 3.09 | M2/M1 |
| 23 | 20654 | IV | 3.14 | M1/M3 |
| 24 | 15232 | IR | 3.27 | M1/M2 |
| 25 | 10116 | IV | 3.40 | M5/M1 |
| 26 | 15693 | IV | 3.43 | M1/M2 |
| 27 | 473 | IV | 3.50 | M1/M4 |
| 28 | 10024 | IV | 3.54 | M3/M3 |
| 29 | 9345 | IV | 3.61 | M4/M1 |
| 30 | 20525 | IV | 3.93 | M2/M4 |
| 31 | 9859 WGS^(5)^ | IV | 4.00 | M1/M1 |
| 32 | 8141 WGS^(5)^ | IV | 4.05 | M1/M2 |
| 33 | 15596 | IV | 4.05 | M1/M5 |
| 34 | 23014 | IR | 4.12 | M4/M4 |
| 35 | OBRG6 | IR | 4.26 | M1/M1 |
| 36 | 4763 | IV | 4.33 | M1/M4 |
| 37 | 8102 WGS | IV | 4.51 | M1/M2 |
| 38 | 20483^(6)^ | IV | 4.58 | M1/M4 |
| 39 | 11360 | IV | 4.83 | M1/M3 |
| 40 | Z860 | IV | 4.97 | M1/M3 |
| 41 | **#Z857** | IV | 5.24 | M4/M3 |
| 42 | OBRF6 | IR | 5.27 | M1/M4 |
| 43 | **#10435** WGS^(5)^ | IV | 5.40 | M1/M1 |
| 44 | **#10465** WGS^(5)^ | IV | 5.56 | M4/rec ^(4)^ |

**Legend of Supplementary Table S1**

1. Six animals for which high quality DNA was available in large quantities were selected for exon capture sequencing: three (in bold and underlined) with a plasma virus load (PVL) among the lowest (below the 25th percentile) and three (in bold and double underlined) among the highest PVL values (above the 75th percentile).
2. Animals were inoculated intrarectally (IR) or intravenously (IV) with SIVmac251.
3. Since PVL at the set point (around 100 days after inoculation) followed a log-normal distribution, we employed the logarithm of the PVL in all calculations.
4. Rec: MHC recombinant haplotype
5. WGS: whole genome sequences of nine animals were characterized in a previous study ^1^
6. All but two animals (#14468 and #20483) were included in our previous study ^1^

**Supplementary Table S2: List of genes studied in six animals.**

| Human GeneSymbol | Description | EnsEmbl ID  (Rhesus macaque genome assembly Mmul_8.0.1) | EnsEmbl ID (Human genome assembly GRCh38.p12) | Length | Exon  Count | Comparison with the list of genes involved in human HIV infection (NCBI database) |
| --- | --- | --- | --- | --- | --- | --- |
| ACTB | actin, beta | ENSMMUT00000015585 |  | 1128 | 5 | Vpr; downregulates |
| ACTG1 | actin, gamma 1 | ENSMMUT00000015586 |  | 1143 | 6 | Vpr; enhances polymerization of actin gamma 1 |
| ADAR | adenosine deaminase, RNA-specific | ENSMMUT00000022632 |  | 3675 | 15 | Tat; downregulates |
| AGFG1 | ArfGAP with FG repeats 1 | ENSMMUT00000044444 |  | 1521 | 12 | Rev; requires |
| AIFM1 | apoptosis-inducing factor, mitochondrion-associated, 1 | ENSMMUT00000006987 |  | 1839 | 16 | Vpr; interacts with |
| AKT1 | v-akt murine thymoma viral oncogene homolog 1 | ENSMMUT00000039437 |  | 1443 | 13 | Vpr; inhibits |
| AKT2 | v-akt murine thymoma viral oncogene homolog 2 | ENSMMUT00000005130 |  | 1446 | 13 | Vpr; inhibits |
| AKT3 | v-akt murine thymoma viral oncogene homolog 3 | ENSMMUT00000044490 |  | 1398 | 13 | Vpr; inhibits |
| ANGPT1 | angiopoietin 1 | ENSMMUT00000024944 |  | 1494 | 9 | HIV-1 viral replication is enhanced by knock-down of human gene |
| ANGPT2 | angiopoietin 2 | ENSMMUT00000014159 |  | 1488 | 9 | absent from NCBI database |
| APOBEC3F | apolipoprotein B mRNA editing enzyme, catalytic polypeptide-like 3F | ENSMMUG00000004017 |  | 1122 | 7 | Vpu; inhibits |
| APOBEC3G | apolipoprotein B mRNA editing enzyme, catalytic polypeptide-like 3G | ENSMMUT00000005690 |  | 1113 | 6 | Vpu; inhibits |
| APOBEC3H | apolipoprotein B mRNA editing enzyme, catalytic polypeptide-like 3H | ENSMMUT00000001449 |  | 630 | 4 | Vif; interacts with |
| APOE | Apolipoprotein E | ENSMMUT00000020072 |  | 768 | 3 | Vpr; upregulates |
| APOL1 | apolipoprotein L, 1 | ENSMMUT00000044038 |  | 1104 | 6 | Vpu; downregulated by |
| ARHGAP32 | Rho GTPase activating protein 32 | ENSMMUT00000004200 |  | 5007 | 17 | HIV-1 viral replication is enhanced by knock-down of human gene |
| ATF2 | activating transcription factor 2 | ENSMMUT00000005599 |  | 1518 | 7 | Nef; inhibits |
| ATF3 | activating transcription factor 3 | ENSMMUT00000045257 |  | 402 | 4 | Tat; upregulates |
| ATM | ataxia telangiectasia mutated | ENSMMUT00000005904 |  | 8853 | 60 | Vpr; activates |
| ATR | ataxia telangiectasia and Rad3 related | ENSMMUT00000011100 |  | 7941 | 49 | Vpr; interacts with |
| ATP5B | ATP synthase, H+ transporting, mitochondrial F1 complex, beta polypeptid | ENSMMUT00000001464 |  | 1587 | 11 | Vpu; interacts with |
| BSG | basigin (Ok blood group) | ENSMMUT00000026339 |  | 1155 | 8 | Tat; downregulates |
| BST2 | bone marrow stromal cell antigen 2 | ENSMMUT00000008172 |  | 546 | 4 | Vpu; relocalizes |
| C2 | complement component 2 | ENSMMUT00000001046 |  | 2256 | 19 | absent from NCBI database |
| C4A | complement component 4A (Rodgers blood group) |  | ENSG00000244731 | 5232 | 41 | absent from NCBI database |
| C4B | complement component 4B  (Chido blood group) |  | ENSG00000224389 | 5232 | 41 | Envelope surface glycoprotein gp120; interacts with |
| CASP1 | caspase 1, apoptosis-related cysteine peptidase  (interleukin 1, beta, convertase) | ENSMMUT00000013767 |  | 789 | 6 | Vpr; activates |
| CASP9 | caspase 9, apoptosis-related cysteine peptidase | ENSMMUT00000047552 |  | 1251 | 9 | Vpr; activates |
| CCL11 | chemokine (C-C motif) ligand 11 | ENSMMUT00000013666 |  | 291 | 3 | Envelope surface glycoprotein gp120; inhibited by |
| CCL13 | chemokine (C-C motif) ligand 13 | ENSMMUT00000013669 |  | 294 | 3 | absent from NCBI database |
| CCL17 | chemokine (C-C motif) ligand 17 | ENSMMUT00000025587 |  | 273 | 3 | absent from NCBI database |
| CCL18 | chemokine (C-C motif) ligand 18 | ENSMMUT00000001338 |  |  | 3 | absent from NCBI database |
| CCL2 | chemokine (C-C motif) ligand 2 | ENSMMUT00000000422 |  | 297 | 3 | Vpr; upregulates |
| CCL20 | chemokine (C-C motif) ligand 20 | ENSMMUT00000018602 |  | 288 | 4 | Tat; upregulates |
| CCL22 | chemokine (C-C motif) ligand 22 |  | ENSG00000102962 | 279 | 3 | Tat; upregulates |
| CCL23 | chemokine (C-C motif) ligand 23 | ENSMMUT00000001337 |  | 360 | 4 | absent from NCBI database |
| CCL25 | chemokine (C-C motif) ligand 25 | ENSMMUT00000014847 |  | 450 | 4 | absent from NCBI database |
| CCL27 | chemokine (C-C motif) ligand 27 | ENSMMUT00000029246 |  | 336 | 3 | HIV-1 viral replication is enhanced by knock-down of human gene |
| CCL28 | chemokine (C-C motif) ligand 28 | ENSMMUT00000000268 |  | 381 | 3 | absent from NCBI database |
| CCL3 | chemokine (C-C motif) ligand 3 | ENSMMUT00000001343 |  | 276 | 3 | Vpr; downregulates |
| CCL4 (CCL4L1) | chemokine (C-C motif) ligand 4 | ENSMMUT00000041948 |  | 393 | 3 | absent from NCBI database |
| CCL5 | chemokine (C-C motif) ligand 5 | ENSMMUT00000006815 |  | 273 | 3 | Vpr; regulates |
| CCL7 | chemokine (C-C motif) ligand 7 | ENSMMUT00000013663 |  | 315 | 3 | Tat; upregulates |
| CCL8 | chemokine (C-C motif) ligand 8 | ENSMMUT00000013668 |  | 297 | 3 | Tat; upregulates |
| CCNT1 | cyclin T1 | ENSMMUT00000010683 |  | 2184 | 9 | Vpr; binds |
| CCR1 | chemokine (C-C motif) receptor 1 | ENSMMUT00000010501 |  | 1065 | 1 | Tat; modulates |
| CCR10 | chemokine (C-C motif) receptor 10 | ENSMMUT00000008515 |  | 1086 | 2 | retropepsin; cleaves |
| CCR2 | chemokine (C-C motif) receptor 2 | ENSMMUT00000020033 |  | 1098 | 2 | Tat; interacts with |
| CCR3 | chemokine (C-C motif) receptor 3 | ENSMMUT00000006470 |  | 1065 | 1 | Tat; upregulates |
| CCR4 | chemokine (C-C motif) receptor 4 | ENSMMUT00000031884 |  | 1080 | 1 | Vif; upregulates |
| CCR5 | chemokine (C-C motif) receptor 5 | ENSMMUG00000042238 |  | 1059 | 1 | Tat; upregulates |
| CCR6 | chemokine (C-C motif) receptor 6 | ENSMMUT00000007979 |  | 1137 | 2 | Envelope surface glycoprotein gp120; inhibits |
| CCR7 | chemokine (C-C motif) receptor 7 | ENSMMUT00000002963 |  | 1134 | 3 | Vif; upregulates |
| CCR8 | chemokine (C-C motif) receptor 8 | ENSMMUT00000032322 |  | 1065 | 1 | Envelope transmembrane glycoprotein gp41; interacts with |
| CCR9 | chemokine (C-C motif) receptor 9 | ENSMMUT00000033183 |  | 1107 | 2 | Vif; downregulates |
| CCRL1 | chemokine (C-C motif) receptor-like 1 | ENSMMUT00000000770 |  | 1050 | 1 | absent from NCBI database |
| CD14 | CD14 molecule | ENSMMUT00000013971 |  | 1125 | 2 | Nef; upregulates |
| CD151 | CD151 molecule  (Raph blood group) | ENSMMUT00000029017 |  | 759 | 7 | Envelope surface glycoprotein gp120; downregulates |
| CD209 | CD209 molecule | ENSMMUT00000031592 |  | 1218 | 7 | Nef; upregulates |
| CD244 | CD244 molecule, natural killer cell receptor 2B4 | ENSMMUT00000024367 |  | 1095 | 9 | absent from NCBI database |
| CD247 | CD247 molecule | ENSMMUT00000011397 |  | 438 | 7 | Vpu; interacts with |
| CD27 | CD27 molecule | ENSMMUT00000020014 |  | 780 | 6 | Envelope surface glycoprotein gp160, precursor; upregulates |
| CD274 | CD274 molecule | ENSMMUT00000017136 |  | 870 | 6 | Tat; upregulates |
| CD28 | CD28 molecule | ENSMMUT00000008415 |  | 660 | 4 | Vpr; downregulates |
| CD4 | CD4 molecule | ENSMMUT00000018518 |  | 1374 | 9 | Vpu; regulates |
| CD40 | CD40 molecule, TNF receptor superfamily member 5 | ENSMMUT00000005213 |  | 714 | 8 | Vpu; upregulates |
| CD40LG | CD40 ligand | ENSMMUT00000019340 |  | 783 | 5 | Tat; synergizes with |
| CD44 | CD44 molecule  (Indian blood group) | ENSMMUT00000006144 |  | 2097 | 17 | Vpr; upregulates |
| CD5 | CD5 molecule | ENSMMUT00000021055 |  | 1407 | 8 | Nef; interacts with |
| CD55 | CD55 molecule, decay accelerating factor for complement  (Cromer blood group) | ENSMMUT00000016407 |  | 1029 | 9 | Pr55(Gag); recruits |
| CD69 | CD69 molecule | ENSMMUT00000021448 |  | 600 | 5 | Vpr; activates |
| CD74 | CD74 molecule, major histocompatibility complex, class II invariant chain | ENSMMUT00000012750 |  | 807 | 7 | Vpu; interacts with |
| CD80 | CD80 molecule | ENSMMUT00000046924 |  | 864 | 5 | Vpr; downregulates |
| CD86 | CD86 molecule | ENSMMUT00000001306 |  | 984 | 7 | Vpr; downregulates |
| CD8A | CD8a molecule | ENSMMUT00000004986 |  | 705 | 6 | Pol; inhibits |
| CD8B | CD8b molecule | ENSMMUT00000004994 |  | 735 | 7 | Pol; inhibits |
| CDC25C | cell division cycle 25 homolog C  (S. pombe) | ENSMMUT00000012608 |  | 1422 | 13 | Vpr; relocalizes |
| CFB | complement factor B | ENSMMUT00000001051 |  | 2289 | 18 | absent from NCBI database |
| CHUK | conserved helix-loop-helix ubiquitous kinase | ENSMMUT00000025976 |  | 2238 | 21 | Tat; relocalizes |
| CIITA | class II, major histocompatibility complex, transactivator | ENSMMUT00000039568 |  | 3393 | 19 | Tat; inhibits |
| CISH | cytokine inducible SH2-containing protein | ENSMMUT00000014688 |  | 774 | 3 | Tat; upregulates |
| CLEC4M | C-type lectin domain family 4, member M |  | ENSG00000104938 | 1200 | 7 | Envelope surface glycoprotein gp120; binds |
| CPT2 | carnitine palmitoyltransferase 2 | ENSMMUT00000010644 |  | 1977 | 5 | Envelope surface glycoprotein gp160, precursor; palmitoylated by |
| CR1 | complement component (3b/4b) receptor 1 (Knops blood group) |  | ENSG00000203710 | 6117 | 39 | Tat; downregulates |
| CREBBP | CREB binding protein | ENSMMUT00000003238 |  | 7326 | 31 | Vpr; binds |
| CTLA4 | cytotoxic T-lymphocyte-associated protein 4 | ENSMMUT00000022346 |  | 669 | 4 | Vpr; upregulates |
| CTSG | cathepsin G | ENSMMUT00000006262 |  | 765 | 5 | absent from NCBI database |
| CUL5 | cullin 5 | ENSMMUT00000002530 |  | 2340 | 19 | Vif; requires |
| CX3CR1 | chemokine (C-X3-C) receptor 1 | ENSMMUT00000032321 |  | 1164 | 3 | Tat; upregulates |
| CXCL10 | chemokine (C-X-C motif) ligand 10 | ENSMMUT00000029391 |  | 294 | 4 | Tat; upregulates |
| CXCL11 | chemokine (C-X-C motif) ligand 11 | ENSMMUT00000042212 |  | 282 | 4 | absent from NCBI database |
| CXCL12 | chemokine (C-X-C motif) ligand 12 (stromal cell-derived factor 1) | ENSMMUT00000042940 |  | 357 | 4 | Tat; upregulates |
| CXCL6 | chemokine (C-X-C motif) ligand 6 (granulocyte chemotactic protein 2) | ENSMMUT00000025054 |  | 324 | 3 | Envelope transmembrane glycoprotein gp41; upregulates |
| CXCR1 (IL8RA) | chemokine (C-X-C motif) receptor 1 | ENSMMUT00000027182 |  | 1053 | 1 | absent from NCBI database |
| CXCR2 | chemokine (C-X-C motif) receptor 2 | ENSMMUT00000018796 |  | 1077 | 1 | matrix; upregulates |
| CXCR3 | chemokine (C-X-C motif) receptor 3 | ENSMMUT00000047946 |  | 1077 | 1 | Vif; upregulates |
| CXCR4 | chemokine (C-X-C motif) receptor 4 | ENSMMUT00000043066 |  | 1053 | 3 | Tat; upregulates |
| CXCR5 | chemokine (C-X-C motif) receptor 5 | ENSMMUT00000013019 |  | 1116 | 2 | absent from NCBI database |
| CXCR6 | chemokine (C-X-C motif) receptor 6 | ENSMMUT00000033182 |  | 1029 | 1 | Envelope surface glycoprotein gp120; interacts with |
| CYCS | cytochrome c, somatic | ENSMMUT00000042168 |  | 318 | 2 | Vpr; induces release of |
| DARC | Duffy blood group, chemokine receptor | ENSMMUT00000001841 |  | 1005 | 2 | absent from NCBI database |
| DDX3X | DEAD (Asp-Glu-Ala-Asp) box polypeptide 3, X-linked | ENSMMUT00000024715 |  | 1911 | 16 | Tat; regulated by |
| DDX39B | DEAD (Asp-Glu-Ala-Asp) box polypeptide 39B | ENSMMUT00000041076 |  | 1287 | 10 | Pr55(Gag); complexes with |
| DDX53 | DEAD (Asp-Glu-Ala-Asp) box polypeptide 53 | ENSMMUT00000031145 |  | 1791 | 2 | HIV-1 viral replication; enhanced by knock-down of human gene |
| DDX58 | DEAD (Asp-Glu-Ala-Asp) box polypeptide 58 | ENSMMUT00000017446 |  | 2775 | 18 | retropepsin; relocalizes |
| DEFB1 | defensin, beta 1 | ENSMMUT00000005370 |  | 204 | 2 | absent from NCBI database |
| DHX9 | DEAH (Asp-Glu-Ala-His) box polypeptide 9 | ENSMMUT00000007925 |  | 3825 | 27 | Tat; interacts with |
| DMXL1 | Dmx-like 1 | ENSMMUT00000026202 |  | 9075 | 43 | HIV-1 viral replication is enhanced by knock-down of human gene |
| DNAJB1 | DnaJ (Hsp40) homolog, subfamily B, member 1 | ENSMMUT00000003928 |  | 1005 | 3 | Nef; interacts with |
| DYSF | dysferlin, limb girdle muscular dystrophy 2B | ENSMMUT00000010022 |  | 4150 | 56 | Vpr; upregulates |
| EDN1 | endothelin 1 | ENSMMUT00000010145 |  | 633 | 5 | Tat; upregulates |
| EIF2AK1 | eukaryotic translation initiation factor 2-alpha kinase 1 | ENSMMUT00000042501 |  | 1890 | 15 | absent from NCBI database |
| EIF2AK2 | eukaryotic translation initiation factor 2-alpha kinase 2 | ENSMMUG00000037522 |  | 1656 | 15 | Vpu; inhibits |
| EIF2AK3 | eukaryotic translation initiation factor 2-alpha kinase 3 | ENSMMUT00000012933 |  | 3348 | 17 | Tat; induces phosphorylation of |
| EIF2AK4 | eukaryotic translation initiation factor 2 alpha kinase 4 |  | ENSG00000128829 | 4950 | 39 | retropepsin; cleaves |
| FAS | Fas (TNF receptor superfamily, member 6) | ENSMMUT00000015642 |  | 1002 | 9 | Vpu; interacts with |
| FASLG | Fas ligand (TNF superfamily, member 6) | ENSMMUT00000010180 |  | 840 | 4 | Tat; upregulates |
| FCAR (CD89) | Fc fragment of IgA | ENSMMUT00000038348 |  | 861 | 5 | absent from NCBI database |
| FCGR1A (CD64) | Fc fragment of IgG, high affinity Ia, receptor (CD64) | ENSMMUT00000006198 |  | 1119 | 6 | absent from NCBI database |
| FCGR2A | Fc fragment of IgG, low affinity IIa, receptor (CD32) | ENSMMUG00000003775 |  | 954 | 7 | Nef; downregulates |
| FCGR2B | Fc fragment of IgG, low affinity IIb, receptor (CD32) |  | ENSG00000072694 |  | 8 | Nef; downregulates |
| FCGR3A (CD16) | Fc fragment of IgG, low affinity IIIa, receptor (CD16a) | ENSMMUT00000022769 |  | 870 | 5 | absent from NCBI database |
| FDPS | farnesyl diphosphate synthase | ENSMMUT00000032559 |  | 1260 | 10 | absent from NCBI database |
| FGF2 | fibroblast growth factor 2 (basic) | ENSMMUT00000010367 |  | 504 | 3 | Tat; upregulates |
| FOXP3 | forkhead box P3 | ENSMMUT00000012057 |  | 1290 | 12 | Tat; upregulates |
| FURIN | furin (paired basic amino acid cleaving enzyme) |  | ENSG00000140564 | 2385 | 15 | Tat; cleaved by |
| FYN | FYN oncogene related to SRC, FGR, YES | ENSMMUT00000039959 |  | 1611 | 11 | Pr55(Gag); enhanced by |
| GATA3 | GATA binding protein 3 | ENSMMUT00000009704 |  | 1332 | 5 | Nef; binds |
| GML | glycosylphosphatidylinositol anchored molecule like protein | ENSMMUT00000019130 |  | 474 | 3 | HIV-1 viral replication is enhanced by knock-down of human gene |
| GNLY | granulysin |  | ENSG00000115523 | 435 | 5 | absent from NCBI database |
| GSK3B | glycogen synthase kinase 3 beta | ENSMMUT00000002800 |  | 1316 | 12 | Vpu; binds |
| GYPA | glycophorin A (MNS blood group) | ENSMMUT00000024847 |  | 201 | 3 | Tat; downregulates |
| GZMA | granzyme A (granzyme 1, cytotoxic T-lymphocyte-associated serine esterase 3) | ENSMMUT00000002260 |  | 786 | 5 | Nef; upregulates |
| GZMB | granzyme B (granzyme 2, cytotoxic T-lymphocyte-associated serine esterase 1) | ENSMMUT00000020179 |  | 843 | 5 | Vif; upregulates |
| GZMK | granzyme K (granzyme 3; tryptase II) | ENSMMUT00000028382 |  | 792 | 5 | absent from NCBI database |
| GZMM | granzyme M (lymphocyte met-ase 1) |  | ENSG00000197540 | 771 | 5 | absent from NCBI database |
| HSP90AA1 | heat shock protein 90kDa alpha (cytosolic), class A member 1 |  | ENSG00000080824 | 2199 | 10 | Tat; upregulates |
| HTATSF1 | HIV-1 Tat specific factor 1 | ENSMMUT00000009046 |  | 2262 | 9 | Tat; stimulated by |
| ICAM1 | intercellular adhesion molecule 1 | ENSMMUT00000013055 |  | 1596 | 7 | Tat; upregulates |
| ICAM3 | intercellular adhesion molecule 3 | ENSMMUT00000027708 |  | 1635 | 7 | Envelope surface glycoprotein gp120; interacts with |
| ICOS | inducible T-cell co-stimulator | ENSMMUT00000003773 |  | 597 | 4 | absent from NCBI database |
| ICOSLG | inducible T-cell co-stimulator ligand | ENSMMUT00000014893 |  | 906 | 5 | absent from NCBI database |
| IFIH1 | interferon induced with helicase C domain 1 | ENSMMUT00000004537 |  | 3075 | 16 | absent from NCBI database |
| IFIT1 | interferon-induced protein with tetratricopeptide repeats 1 | ENSMMUT00000024656 |  | 1434 | 2 | Vpu; inhibits |
| IFIT2 | interferon-induced protein with tetratricopeptide repeats 2 | ENSMMUT00000005945 |  | 1416 | 2 | Vpu; inhibits |
| IFIT3 | interferon-induced protein with tetratricopeptide repeats 3 | ENSMMUT00000007046 |  | 1458 | 1 | Vpu; inhibits |
| IFITM1 | interferon induced transmembrane protein 1 | ENSMMUT00000018611 |  | 375 | 2 | Vif; downregulated by |
| IFITM2 | interferon induced transmembrane protein 2 |  | ENSG00000185201 |  |  | Vif; downregulated by |
| IFITM3 | interferon induced transmembrane protein 3 | ENSMMUT00000018609 |  | 399 | 2 | Vif; downregulated by |
| IFNA1 | interferon, alpha 1 | ENSMMUT00000046270 |  | 567 | 1 | Vpu; interacts with |
| IFNA14 | interferon, alpha 14 | ENSMMUT00000046268 |  | 567 | 1 | Vpu; interacts with |
| IFNA16 | interferon, alpha 16 | ENSMMUT00000015333 |  | 570 | 1 | Vpu; interacts with |
| IFNA2 | interferon, alpha 2 | ENSMMUT00000033242 |  | 564 | 1 | Vpu; interacts with |
| IFNA4 | interferon, alpha 4 | ENSMMUT00000046266 |  | 567 | 1 | Vpu; interacts with |
| IFNA8 | interferon, alpha 8 | ENSMMUT00000026686 |  | 567 | 1 | Vpu; interacts with |
| IFNAR1 | interferon (alpha, beta and omega) receptor 1 | ENSMMUT00000005618 |  | 1680 | 11 | Envelope surface glycoprotein gp120; upregulates |
| IFNAR2 | interferon (alpha, beta and omega) receptor 2 | ENSMMUT00000005614 |  | 996 | 8 | Tat; upregulates |
| IFNB1 | interferon, beta 1, fibroblast | ENSMMUT00000026788 |  | 561 | 1 | Vpu; inhibits |
| IFNG | interferon, gamma | ENSMMUT00000027007 |  | 495 | 4 | Vpr; upregulates |
| IFNGR1 | interferon gamma receptor 1 | ENSMMUT00000016941 |  | 1464 | 7 | absent from NCBI database |
| IFNGR2 | interferon gamma receptor 2 (interferon gamma transducer 1) | ENSMMUT00000007749 |  | 1008 | 8 | Envelope surface glycoprotein gp120; upregulates |
| IFNW1 | interferon, omega 1 | ENSMMUT00000026787 |  | 585 | 1 | Envelope transmembrane glycoprotein gp41; upregulates |
| IGF1 | insulin-like growth factor 1 (somatomedin C) | ENSMMUT00000003166 |  | 588 | 4 | Tat; upregulates |
| IKBKB | inhibitor of kappa light polypeptide gene enhancer in B-cells, kinase beta | ENSMMUT00000011677 |  | 2271 | 21 | Tat; induces phosphorylation of |
| IKBKE | inhibitor of kappa light polypeptide gene enhancer in B-cells, kinase epsilon | ENSMMUT00000045173 |  | 1974 | 18 | Envelope surface glycoprotein gp120; activates |
| IL10 | interleukin 10 | ENSMMUT00000033151 |  | 534 | 5 | Vpr; upregulates |
| IL10RA | interleukin 10 receptor, alpha | ENSMMUT00000010671 |  | 1668 | 6 | absent from NCBI database |
| IL10RB | interleukin 10 receptor, beta | ENSMMUT00000005616 |  | 804 | 6 | absent from NCBI database |
| IL11RA | interleukin 11 receptor, alpha | ENSMMUT00000029244 |  | 1266 | 12 | absent from NCBI database |
| IL12A | interleukin 12A (natural killer cell stimulatory factor 1, cytotoxic lymphocyte maturation factor 1, p35) | ENSMMUT00000032477 |  | 759 | 7 | Vpr; modulates |
| IL12B | interleukin 12B (natural killer cell stimulatory factor 2, cytotoxic lymphocyte maturation factor 2, p40) | ENSMMUT00000030533 |  | 984 | 6 | Tat; upregulates |
| IL12RB1 | interleukin 12 receptor, beta 1 | ENSMMUT00000016938 |  | 1752 | 13 | absent from NCBI database |
| IL12RB2 | interleukin 12 receptor, beta 2 | ENSMMUT00000000941 |  | 2589 | 15 | absent from NCBI database |
| IL13 | interleukin 13 | ENSMMUT00000004436 |  | 459 | 3 | Tat; induces release of |
| IL13RA1 | interleukin 13 receptor, alpha 1 | ENSMMUT00000016116 |  | 663 | 5 | absent from NCBI database |
| IL15 | interleukin 15 | ENSMMUT00000026825 |  | 483 | 5 | Nef; upregulates |
| IL15RA | interleukin 15 receptor, alpha | ENSMMUT00000021237 |  | 798 | 7 | absent from NCBI database |
| IL16 | interleukin 16 (lymphocyte chemoattractant factor) | ENSMMUT00000016182 |  | 3993 | 18 | Tat; inhibited by |
| IL17A | interleukin 17 receptor A | ENSMMUT00000010352 |  | 465 | 3 | Tat; upregulates |
| IL17B | interleukin 17 receptor B | ENSMMUT00000030489 |  | 540 | 3 | absent from NCBI database |
| IL17RA | interleukin 17 receptor A | ENSMMUT00000044342 |  | 2595 | 13 | absent from NCBI database |
| IL17RB | interleukin 17 receptor B | ENSMMUT00000027060 |  | 1506 | 11 | absent from NCBI database |
| IL17RC | interleukin 17 receptor C | ENSMMUT00000025694 |  | 576 | 6 | absent from NCBI database |
| IL17RD | interleukin 17 receptor D | ENSMMUT00000046455 |  | 1785 | 9 | absent from NCBI database |
| IL17RE | interleukin 17 receptor E | ENSMMUT00000025690 |  | 1995 | 16 | absent from NCBI database |
| IL18 | interleukin 18 | ENSMMUT00000021372 |  | 579 | 5 | absent from NCBI database |
| IL18R1 | interleukin 18 receptor 1 | ENSMMUT00000012921 |  | 1620 | 10 | HIV-1 viral replication is enhanced by knock-down of human gene |
| IL18RAP | interleukin 18 receptor accessory protein | ENSMMUT00000043196 |  | 1794 | 10 | absent from NCBI database |
| IL19 | interleukin 19 | ENSMMUT00000033156 |  | 531 | 5 | Tat; upregulates |
| IL1A | interleukin 1, alpha | ENSMMUT00000027798 |  | 813 | 6 | Tat; upregulates |
| IL1B | interleukin 1, beta | ENSMMUT00000027799 |  | 804 | 6 | Vpr; activates |
| IL1F10 | interleukin 1 family, member 10 (theta) | ENSMMUT00000043102 |  | 489 | 4 | absent from NCBI database |
| IL1F5 | interleukin 1 family, member 5 (delta) | ENSMMUT00000025593 |  | 465 | 4 | absent from NCBI database |
| IL37 (Il1F7) | interleukin 1 family, member 7  (zeta) | ENSMMUT00000008287 |  | 405 | 4 | absent from NCBI database |
| IL1R1 | interleukin 1 receptor-like 1 | ENSMMUT00000029166 |  | 1707 | 10 | Tat; upregulates |
| IL1R2 | interleukin 1 receptor-like 2 | ENSMMUT00000029169 |  | 1182 | 8 | Envelope surface glycoprotein gp120; activates |
| IL1RN | interleukin 1 receptor antagonist | ENSMMUT00000018251 |  | 537 | 6 | Tat; upregulates |
| IL2 | interleukin 2 | ENSMMUT00000029666 |  | 462 | 4 | Vpr; downregulates |
| IL20 | interleukin 20 | ENSMMUT00000033157 |  | 528 | 5 | Tat; upregulates |
| IL21 | interleukin 21 | ENSMMUT00000004728 |  | 486 | 5 | absent from NCBI database |
| IL21R | interleukin 21 receptor | ENSMMUT00000011533 |  | 1461 | 6 | absent from NCBI database |
| IL22 | interleukin 22 | ENSMMUT00000026319 |  | 537 | 5 | absent from NCBI database |
| IL23A | interleukin 23, alpha subunit p19 | ENSMMUT00000046362 |  | 567 | 4 | absent from NCBI database |
| IL25 | interleukin 25 | ENSMMUT00000020692 |  | 528 | 2 | absent from NCBI database |
| IL27 | interleukin 27 | ENSMMUT00000038874 |  | 333 | 5 | absent from NCBI database |
| IL28A | interleukin 28A  (interferon, lambda 2) | ENSMMUT00000026374 |  | 600 | 6 | absent from NCBI database |
| IL28B | interleukin 28B  (interferon, lambda 3) |  | ENSG00000197110 | 588 | 5 | absent from NCBI database |
| IL28RA | interleukin 28 receptor, alpha (interferon, lambda receptor) | ENSMMUT00000032442 |  | 1563 | 8 | absent from NCBI database |
| IL29 | interleukin 29 (interferon, lambda 1) |  | ENSG00000182393 | 600 | 5 | absent from NCBI database |
| IL2RA | interleukin 2 receptor, alpha | ENSMMUT00000043345 |  | 795 | 7 | Tat; upregulates |
| IL2RB | interleukin 2 receptor, beta | ENSMMUT00000007618 |  | 1653 | 9 | Tat; downregulates |
| IL2RG | interleukin 2 receptor, gamma (severe combined immunodeficiency) | ENSMMUT00000025011 |  | 1107 | 8 | Envelope surface glycoprotein gp120; downregulates |
| IL3 | interleukin 3 (colony-stimulating factor, multiple) | ENSMMUT00000023756 |  | 429 | 5 | Tat; upregulates |
| IL33 | interleukin 33 | ENSMMUT00000030043 |  | 714 | 7 | absent from NCBI database |
| IL3RA | interleukin 3 receptor, alpha (low affinity) |  | ENSG00000185291 | 1134 | 11 | absent from NCBI database |
| IL4 | interleukin 4 | ENSMMUT00000004438 |  | 459 | 4 | Vpr; downregulates |
| IL4R | interleukin 4 receptor | ENSMMUT00000006594 |  | 2172 | 10 | Tat; upregulates |
| IL5 | interleukin 5 (colony-stimulating factor, eosinophil) | ENSMMUT00000004434 |  | 402 | 4 | absent from NCBI database |
| IL5RA | interleukin 5 receptor, alpha | ENSMMUT00000002613 |  | 1260 | 10 | absent from NCBI database |
| IL6 | interleukin 6 (interferon, beta 2) | ENSMMUT00000028086 |  | 636 | 5 | Vpr; upregulates |
| IL6R | interleukin 6 receptor | ENSMMUT00000032343 |  | 1404 | 10 | Tat; upregulates |
| IL6ST | interleukin 6 signal transducer (gp130, oncostatin M receptor) | ENSMMUT00000002670 |  | 2571 | 15 | HIV-1 viral replication is enhanced by knock-down of human gene |
| IL7 | interleukin 7 | ENSMMUT00000002348 |  | 525 | 5 | Tat; inhibits |
| IL7R | interleukin 7 receptor | ENSMMUT00000002374 |  | 1377 | 8 | Vif; upregulates |
| IL8 | interleukin 8 | ENSMMUT00000005432 |  | 297 | 4 | absent from NCBI database |
| IL9 | interleukin 9 | ENSMMUT00000002759 |  | 435 | 5 | absent from NCBI database |
| IL9R | interleukin 9 receptor | ENSMMUT00000002856 |  | 1548 | 9 | absent from NCBI database |
| IPO5 | importin 5 | ENSMMUT00000018999 |  | 3345 | 27 | Vpr; regulates |
| IRAK1 | interleukin-1 receptor-associated kinase 1 | ENSMMUT00000028542 |  | 1914 | 14 | absent from NCBI database |
| IRAK4 | interleukin-1 receptor-associated kinase 4 | ENSMMUT00000000302 |  | 1380 | 11 | absent from NCBI database |
| IRF1 | interferon regulatory factor 1 | ENSMMUT00000011322 |  | 975 | 10 | Vif; downregulates |
| IRF3 | interferon regulatory factor 3 | ENSMMUT00000038423 |  | 1678 | 7 | Vpu; interacts with |
| IRF5 | interferon regulatory factor 5 | ENSMMUT00000041831 |  | 1503 | 8 | absent from NCBI database |
| IRF7 | interferon regulatory factor 7 | ENSMMUT00000009923 |  | 1515 | 10 | Tat; upregulates |
| IRF9 | interferon regulatory factor 9 | ENSMMUT00000017496 |  | 1173 | 9 | absent from NCBI database |
| ISG15 | ISG15 ubiquitin-like modifier | ENSMMUT00000002559 |  | 495 | 2 | Vpu; inhibits |
| ITGA1 | integrin, alpha 1 | ENSMMUT00000002476 |  | 3537 | 30 | absent from NCBI database |
| ITGA2 | integrin, alpha 2 (CD49B, alpha 2 subunit of VLA-2 receptor) | ENSMMUT00000028770 |  | 3477 | 29 | absent from NCBI database |
| ITGB1 | integrin, beta 1 (fibronectin receptor, beta polypeptide, antigen CD29 includes MDF2, MSK12) | ENSMMUT00000024977 |  | 2013 | 13 | Vpr; upregulates |
| IVNS1ABP | influenza virus NS1A binding protein | ENSMMUT00000019491 |  | 1929 | 13 | Pr55(Gag); interacts with |
| JAK1 | Janus kinase 1 | ENSMMUT00000026100 |  | 3435 | 23 | Tat; regulated by |
| JAK2 | Janus kinase 2 | ENSMMUT00000020426 |  | 3399 | 23 | absent from NCBI database |
| JAK3 | Janus kinase 3 | ENSMMUT00000015376 |  | 2691 | 19 | Tat; upregulates |
| JUN | proto-oncogene |  | ENSG00000177606 | 996 | 1 | Vpr; activates |
| KEAP1 | kelch-like ECH-associated protein 1 |  | ENSG00000079999 | 1875 | 5 | Tat; regulated by |
| KLRG1 | killer cell lectin-like receptor subfamily G, member 1 | ENSMMUT00000002015 |  | 558 | 6 | absent from NCBI database |
| KPNA1 | karyopherin alpha 1 (importin alpha 5) | ENSMMUT00000022995 |  | 1617 | 13 | Vpr; interacts with |
| KPNA2 | karyopherin alpha 2 (RAG cohort 1, importin alpha 1) | ENSMMUT00000025648 |  | 1590 | 10 | Vpr; interacts with |
| LEF1 | lymphoid enhancer-binding factor 1 | ENSMMUT00000041894 |  | 1197 | 12 | Tat; downregulates |
| LCP2 | lymphocyte cytosolic protein 2 | ENSMMUT00000019827 |  | 1599 | 20 | Nef; inhibits |
| LIG4 | ligase IV, DNA, ATP-dependent | ENSMMUT00000012407 |  | 1734 | 3 | absent from NCBI database |
| LTA | lymphotoxin alpha  (TNF superfamily, member 1) | ENSMMUT00000012357 |  | 615 | 3 | Tat; upregulates |
| LTB | lymphotoxin beta  (TNF superfamily, member 3) | ENSMMUT00000012368 |  | 732 | 4 | absent from NCBI database |
| LYPD4 | LY6/PLAUR domain containing 4 | ENSMMUT00000018833 |  | 738 | 5 | HIV-1 viral replication is enhanced by knock-down of human gene |
| MAP2K1 | mitogen-activated protein kinase kinase 1 | ENSMMUT00000023660 |  | 1182 | 11 | Vpr; cooperates with |
| MAP2K2 | mitogen-activated protein kinase kinase 2 | ENSMMUT00000027482 |  | 903 | 11 | Vpr; downregulates |
| MAP2K3 | mitogen-activated protein kinase kinase 3 | ENSMMUT00000042087 |  | 1044 | 12 | Tat; interacts with |
| MAP2K4 | mitogen-activated protein kinase kinase 4 | ENSMMUT00000027885 |  | 1200 | 11 | absent from NCBI database |
| MAP2K6 | mitogen-activated protein kinase kinase 6 | ENSMMUT00000001498 |  | 1005 | 12 | Tat; interacts with |
| MAP2K7 | mitogen-activated protein kinase kinase 7 | ENSMMUT00000033435 |  | 1203 | 9 | Vpr; activates |
| MAP4 | microtubule-associated protein 4 | ENSMMUG00000003823 |  | 3459 | 18 | HIV-1 viral replication is enhanced by knock-down of human gene |
| MAPK1 | mitogen-activated protein kinase 1 | ENSMMUT00000017561 |  | 1083 | 8 | Vpr; activates |
| MAPK10 | mitogen-activated protein kinase 10 | ENSMMUT00000011669 |  | 1395 | 12 | Envelope surface glycoprotein gp160, precursor; inhibits |
| MAPK11 | mitogen-activated protein kinase 11 | ENSMMUT00000019806 |  | 1095 | 12 | Tat; activates |
| MAPK12 | mitogen-activated protein kinase 12 | ENSMMUT00000019805 |  | 1104 | 12 | reverse transcriptase; interacts with |
| MAPK13 | mitogen-activated protein kinase 13 |  | ENSG00000156711 | 1098 | 12 | Pr55(Gag); interacts with |
| MAPK14 | mitogen-activated protein kinase 14 |  | ENSG00000112062 | 1083 | 12 | Vpr; cooperates with |
| MAPK3 | mitogen-activated protein kinase 3 | ENSMMUT00000024959 |  | 978 | 7 | Vpr; activates |
| MAPK8 | mitogen-activated protein kinase 8 | ENSMMUT00000005767 |  | 1284 | 11 | Vpr; activates |
| MAPK9 | mitogen-activated protein kinase 9 |  | ENSG00000050748 | 1275 | 11 | Pr55(Gag); interacts with |
| MBL1 | mannose-binding lectin (protein C) 1 soluble | ENSMMUT00000003954 |  | 747 | 4 | absent from NCBI database |
| MBL2 | mannose-binding lectin (protein C) 2, soluble | ENSMMUT00000026856 |  | 744 | 4 | Envelope surface glycoprotein gp120; binds |
| MED28 | mediator complex subunit 28 |  | ENSG00000118579 | 537 | 4 | Tat; regulated by |
| MED6 | mediator complex subunit 6 | ENSMMUT00000008112 |  | 738 | 8 | Tat; regulated by |
| MIF | macrophage migration inhibitory factor (glycosylation-inhibiting factor) | ENSMMUT00000012742 |  | 345 | 3 | Vpr; upregulates |
| MMP9 | matrix metallopeptidase 9 (gelatinase B, 92kDa gelatinase, 92kDa type IV collagenase) | ENSMMUT00000023244 |  | 2121 | 13 | Tat; upregulates |
| MX1 | myxovirus (influenza virus) resistance 1, interferon-inducible protein p78 (mouse) | ENSMMUT00000021494 |  | 1983 | 13 | Vpu; inhibits |
| MYD88 | myeloid differentiation primary response gene (88) | ENSMMUT00000001422 |  | 891 | 5 | Vpr; cooperates with |
| NCR1 | natural cytotoxicity triggering receptor 1 | ENSMMUT00000009189 |  | 687 | 5 | Envelope surface glycoprotein gp120; downregulates |
| NEMO | inhibitor of kappa light polypeptide gene enhancer in B-cells, kinase gamma | ENSMMUT00000047719 |  | 1464 | 10 | absent from NCBI database |
| NFE2L2 | nuclear factor (erythroid-derived 2)-like 2 | ENSMMUT00000002623 |  | 1818 | 5 | Envelope surface glycoprotein gp120; upregulates |
| NFKB1 | nuclear factor of kappa light polypeptide gene enhancer in B-cells 1 | ENSMMUT00000041936 |  | 2907 | 23 | Vpu; inhibits |
| NFKB2 | nuclear factor of kappa light polypeptide gene enhancer in B-cells 2 | ENSMMUT00000004237 |  | 2577 | 21 | Vpr; induces phosphorylation of |
| NFKBIA | nuclear factor of kappa light polypeptide gene enhancer in B-cells inhibitor, alpha | ENSMMUT00000028654 |  | 954 | 6 | Vpu; stabilizes |
| NFKBIB | nuclear factor of kappa light polypeptide gene enhancer in B-cells inhibitor, beta | ENSMMUT00000033265 |  | 1071 | 6 | Vpr; upregulates |
| NFKBIL1 | nuclear factor of kappa light polypeptide gene enhancer in B-cells inhibitor-like 1 | ENSMMUT00000012353 |  | 1143 | 4 | absent from NCBI database |
| NLRP3 | NLR family, pyrin domain containing 3 | ENSMMUG00000005946 |  | 3111 | 9 | absent from NCBI database |
| NLRX1 | NLR family member X1 | ENSMMUT00000017067 |  | 2919 | 9 | HIV-1 viral replication is enhanced by knock-down of human gene |
| NOD2 | nucleotide-binding oligomerization domain containing 2 | ENSMMUT00000017096 |  | 3132 | 12 | absent from NCBI database |
| NOS2 | nitric oxide synthase 2, inducible | ENSMMUT00000014078 |  | 3459 | 28 | Vpr; upregulates |
| NUP153 | nucleoporin 153kDa | ENSMMUT00000002823 |  | 4422 | 22 | Vpr; interacts with |
| NUP85 | nucleoporin 85kDa |  | ENSG00000125450 | 1971 | 19 | Vpr; induces accumulation of |
| NUP98 | nucleoporin 98kDa |  | ENSG00000110713 | 5403 | 32 | Tat; interacts with |
| NXF1 | nuclear RNA export factor 1 | ENSMMUT00000000280 |  | 1860 | 22 | Pr55(Gag); regulated by |
| OAS1 | 2'-5'-oligoadenylate synthetase 1, 40/46kDa | ENSMMUT00000017932 |  | 1203 | 6 | Tat; interacts with |
| OAS2 | 2'-5'-oligoadenylate synthetase 2, 69/71kDa | ENSMMUT00000011884 |  | 2160 | 11 | Tat; interacts with |
| OAS3 | 2'-5'-oligoadenylate synthetase 3, 100kDa |  | ENSG00000111331 | 3264 | 16 | Tat; interacts with |
| OASL | 2'-5'-oligoadenylate synthetase-like | ENSMMUT00000009036 |  | 1548 | 6 | HIV-1 viral replication is enhanced by knock-down of human gene |
| PABPN1 | poly(A) binding protein, nuclear 1 |  | ENSG00000100836 | 921 | 7 | Pr55(Gag); complexes with |
| PARP1 | poly (ADP-ribose) polymerase 1 | ENSMMUT00000030705 |  | 3042 | 23 | Vpr; recruits |
| PDCD1 | programmed cell death 1 | ENSMMUT00000012015 |  | 531 | 3 | Pr55(Gag); interacts with |
| PML | promyelocytic leukemia | ENSMMUT00000003654 |  | 2649 | 8 | Tat; regulated by |
| PPIA | peptidylprolyl isomerase A (cyclophilin A) | ENSMMUT00000023383 |  | 495 | 5 | Vpr; isomerized by |
| PPP2R2B | protein phosphatase 2, regulatory subunit B, beta | ENSMMUT00000028806 |  | 1527 | 10 | Vpr; upregulates |
| PRF1 | perforin 1 (pore forming protein) | ENSMMUT00000008960 |  | 1665 | 2 | Tat; downregulates |
| PRKCA | protein kinase C, alpha | ENSMMUT00000025827 |  | 2019 | 17 | Tat; regulated by |
| PRKCB | protein kinase C, beta | ENSMMUT00000020024 |  | 2022 | 17 | Tat; regulated by |
| PSMB8 | proteasome (prosome, macropain) subunit, beta type, 8 (large multifunctional peptidase 7) | ENSMMUT00000015819 |  | 828 | 6 | Vif; interacts with |
| PSMB9 | proteasome (prosome, macropain) subunit, beta type, 9 (large multifunctional peptidase 2) | ENSMMUT00000015822 |  | 657 | 6 | Vif; interacts with |
| PTPRC | protein tyrosine phosphatase, receptor type, C | ENSMMUT00000000016 |  | 3837 | 30 | Tat; interacts with |
| RAF1 | v-raf-1 murine leukemia viral oncogene homolog 1 | ENSMMUT00000010416 |  | 1947 | 16 | Nef; binds |
| RANBP1 | RAN binding protein 1 | ENSMMUT00000028276 |  | 600 | 6 | Vpu; interacts with |
| RANBP2 | RAN binding protein 2 |  | ENSG00000153201 | 9675 | 29 | Tat; regulated by |
| RAP1A | RAP1A, member of RAS oncogene family | ENSMMUT00000020321 |  | 552 | 6 | capsid; downregulated by |
| RARB | retinoic acid receptor, beta | ENSMMUT00000010300 |  | 1368 | 12 | Nef; inhibits |
| RELA | v-rel reticuloendotheliosis viral oncogene homolog A (avian) | ENSMMUT00000044528 |  | 1632 | 10 | Vpu; inhibited by |
| RNASEL | ribonuclease L (2',5'-oligoisoadenylate synthetase-dependent) | ENSMMUT00000007923 |  | 2226 | 6 | HIV-1 viral replication; enhanced by knock-down of human gene |
| RORA | RAR-related orphan receptor A | ENSMMUT00000025578 |  | 1404 | 10 | absent from NCBI database |
| RORB | RAR-related orphan receptor B | ENSMMUT00000016998 |  | 1377 | 10 | Vif; upregulates |
| RORC | RAR-related orphan receptor C | ENSMMUT00000017000 |  | 1554 | 11 | absent from NCBI database |
| RSAD2 | radical S-adenosyl methionine domain containing 2 | ENSMMUT00000022457 |  | 1083 | 6 | Vpu; inhibits |
| SELL | selectin L | ENSMMUT00000023282 |  | 1125 | 7 | Envelope surface glycoprotein gp120; downregulates |
| SLC14A1 | solute carrier family 14  (urea transporter), member 1  (Kidd blood group) | ENSMMUT00000015430 |  | 1116 | 10 | Tat; downregulates |
| SLC4A1 | solute carrier family 4, anion exchanger, member 1 (erythrocyte membrane protein band 3, Diego blood group) | ENSMMUT00000024043 |  | 2433 | 20 | HIV-1 viral replication is enhanced by knock-down of human gene |
| SLPI | secretory leukocyte peptidase inhibitor | ENSMMUT00000014459 |  | 396 | 4 | retropepsin; inhibited by |
| SOCS3 | suppressor of cytokine signaling 3 | ENSMMUT00000024640 |  | 675 | 1 | Tat; upregulates |
| SPN | sialophorin | ENSMMUT00000022693 |  | 1179 | 4 | Pr55(Gag); co-localizes with |
| STAT1 | signal transducer and activator of transcription 1, 91kDa | ENSMMUT00000007897 |  | 2250 | 23 | Vpu; inhibits |
| STAT2 | signal transducer and activator of transcription 2, 113kDa | ENSMMUT00000001167 |  | 2556 | 23 | Nef; induces phosphorylation of |
| STAT4 | signal transducer and activator of transcription 4 | ENSMMUT00000007899 |  | 2223 | 22 | absent from NCBI database |
| STAT5A | signal transducer and activator of transcription 5A | ENSMMUT00000014971 |  | 2382 | 18 | Envelope surface glycoprotein gp120; downregulates |
| STAT5B | signal transducer and activator of transcription 5B | ENSMMUT00000004862 |  | 1170 | 8 | Envelope surface glycoprotein gp120; downregulates |
| STAT6 | signal transducer and activator of transcription 6, interleukin-4 induced | ENSMMUT00000024650 |  | 2541 | 21 | Tat; interacts with |
| STAU2 | staufen, RNA binding protein, homolog 2 (Drosophila) | ENSMMUT00000030384 |  | 1710 | 12 | Pr55(Gag); interacts with |
| SYK | spleen tyrosine kinase | ENSMMUT00000002471 |  | 1905 | 13 | Pr55(Gag); interacts with |
| TAP1 | transporter 1, ATP-binding cassette, sub-family B (MDR/TAP) | ENSMMUT00000015816 |  | 2424 | 11 | Tat; upregulates |
| TAP2 | transporter 2, ATP-binding cassette, sub-family B (MDR/TAP) | ENSMMUT00000015799 |  | 2103 | 14 | absent from NCBI database |
| TAPBP | TAP binding protein (tapasin) | ENSMMUT00000005492 |  | 1344 | 8 | absent from NCBI database |
| TBK1 | TANK-binding kinase 1 (NF-kB-activating kinase) | ENSMMUT00000010727 |  | 2190 | 20 | absent from NCBI database |
| TBX21 | T-box 21 | ENSMMUT00000009906 |  | 1605 | 6 | Vif; downregulates |
| TCEB3 | transcription elongation factor B (SIII) polypeptide 3 (110kDa, elongin A) | ENSMMUT00000004493 |  | 2250 | 10 | Tat; interacts with |
| TCF4 | transcription factor 4 | ENSMMUT00000016803 |  | 2013 | 18 | Tat; regulated by |
| TGFA | transforming growth factor, alpha | ENSMMUT00000014813 |  | 486 | 6 | Tat; upregulates |
| TGFB1 | transforming growth factor, beta 1 |  | ENSG00000105329 | 1173 | 7 | Vpr; modulates |
| TICAM1 | toll-like receptor adaptor molecule 1 | ENSMMUT00000038871 |  | 2133 | 2 | absent from NCBI database |
| TIRAP | toll-interleukin 1 receptor (TIR) domain containing adaptor protein | ENSMMUT00000011117 |  | 660 | 5 | absent from NCBI database |
| TLR2 | toll-like receptor 2 | ENSMMUT00000022268 |  | 2352 | 1 | Tat; upregulates |
| TLR3 | toll-like receptor 3 | ENSMMUT00000030621 |  | 2712 | 4 | absent from NCBI database |
| TLR4 | toll-like receptor 4 | ENSMMUT00000046758 |  | 2493 | 4 | Vpr; downregulates |
| TLR7 | toll-like receptor 7 | ENSMMUT00000020000 |  | 3147 | 2 | reverse transcriptase; inhibited by |
| TLR8 | toll-like receptor 8 | ENSMMUT00000020002 |  | 3174 | 2 | absent from NCBI database |
| TLR9 | toll-like receptor 9 | ENSMMUT00000017175 |  | 3096 | 2 | Tat; downregulates |
| TNF | tumor necrosis factor (TNF superfamily, member 2) | ENSMMUT00000012362 |  | 699 | 4 | Vpu; inhibits |
| TNFRSF10A | tumor necrosis factor receptor superfamily, member 10a | ENSMMUT00000023399 |  | 1110 | 9 | Envelope surface glycoprotein gp120; upregulates |
| TNFRSF10B | tumor necrosis factor receptor superfamily, member 10b |  | ENSG00000120889 | 1323 | 9 | Envelope surface glycoprotein gp120; upregulates |
| TNFRSF10C | tumor necrosis factor receptor superfamily, member 10c |  | ENSG00000173535 | 780 | 5 | Envelope surface glycoprotein gp120; upregulates |
| TNFRSF10D | tumor necrosis factor receptor superfamily, member 10d, decoy with truncated death domain | ENSMMUT00000023397 |  | 1146 | 9 | Vif; upregulates |
| TNFRSF11A | tumor necrosis factor receptor superfamily, member 11a, NFKB activator | ENSMMUT00000003128 |  | 447 | 4 | absent from NCBI database |
| TNFRSF11B | tumor necrosis factor receptor superfamily, member 11b | ENSMMUT00000023150 |  | 1284 | 5 | absent from NCBI database |
| TNFRSF19 | tumor necrosis factor receptor superfamily, member 19 | ENSMMUT00000006012 |  | 1104 | 7 | absent from NCBI database |
| TNFRSF1A | tumor necrosis factor receptor superfamily, member 1A | ENSMMUT00000000541 |  | 1344 | 9 | Tat; upregulates |
| TNFRSF9 | tumor necrosis factor receptor superfamily, member 9 | ENSMMUT00000023238 |  | 762 | 7 | Tat; upregulates |
| TNFSF10 | tumor necrosis factor (ligand) superfamily, member 10 | ENSMMUT00000027108 |  | 843 | 5 | Vif; downregulates |
| TRAF3 | TNF receptor-associated factor 3 | ENSMMUT00000016459 |  | 1653 | 10 | Tat; downregulates |
| TRAF6 | TNF receptor-associated factor 6 | ENSMMUT00000025667 |  | 1575 | 7 | Nef; requires |
| TRIM10 | tripartite motif-containing 10 | ENSMMUT00000016985 |  | 1182 | 10 | absent from NCBI database |
| TRIM21 | tripartite motif-containing 21 | ENSMMUT00000007012 |  | 1425 | 6 | Pr55(Gag); interacts with |
| TRIM22 | tripartite motif-containing 22 | ENSMMUT00000000496 |  | 1494 | 7 | Tat; upregulates |
| TRIM25 | tripartite motif-containing 25 | ENSMMUT00000008123 |  | 1890 | 9 | Pr55(Gag); interacts with |
| TRIM27 | tripartite motif-containing 27 | ENSMMUT00000022440 |  | 1539 | 8 | HIV-1 viral replication is inhibited by knock-down of human gene |
| TRIM32 | tripartite motif-containing 32 | ENSMMUT00000018383 |  | 1959 | 1 | Tat; binds |
| TRIM37 | tripartite motif-containing 37 | ENSMMUT00000016018 |  | 2889 | 25 | absent from NCBI database |
| TRIM5 | tripartite motif-containing 5 | ENSMMUT00000000490 |  | 1491 | 7 | Vpu; inhibits |
| TSG101 | tumor susceptibility gene 101 | ENSMMUT00000020492 |  | 1149 | 12 | Pr55(Gag); regulated by |
| TYK2 | non-receptor tyrosine kinase | ENSMMUT00000004400 |  | 1175 | 26 | absent from NCBI database |
| UNG | uracil-DNA glycosylase | ENSMMUT00000009721 |  | 939 | 7 | Vpr; interacts with |
| USP18 | ubiquitin specific peptidase 18 | ENSMMUT00000004628 |  | 1122 | 10 | absent from NCBI database |
| VDAC1 | voltage-dependent anion channel 1 | ENSMMUT00000022810 |  | 852 | 8 | Vpr; interacts with |
| VDR | vitamin D (1,25- dihydroxyvitamin D3) receptor | ENSMMUT00000009414 |  | 1302 | 9 | HIV-1 viral replication is enhanced by knock-down of human gene |
| VEGFA | vascular endothelial growth factor A | ENSMMUT00000006471 |  | 1167 | 8 | Tat; upregulates |
| VEGFB | vascular endothelial growth factor B | ENSMMUT00000t018151 |  | 621 | 6 | Tat; upregulates |
| VEGFC | vascular endothelial growth factor C | ENSMMUT00000005304 |  | 1260 | 7 | Tat; upregulates |
| XCL1 | chemokine (C motif) ligand 1 | ENSMMUT00000014080 |  | 1227 | 3 | Tat; upregulates |
| XCR1 | chemokine (C motif) receptor 1 | ENSMMUT00000033177 |  | 999 | 1 | absent from NCBI database |
| XPO1 | exportin 1 (CRM1 homolog, yeast) | ENSMMUT00000043531 |  | 3216 | 24 | Vpu; interacts with |
| XRCC5 | X-ray repair complementing defective repair in Chinese hamster cells 5 (double-strand-break rejoining) | ENSMMUT00000002733 |  | 1206 | 13 | Tat; interacts with |
| YWHAH | tyrosine 3-monooxygenase/tryptophan 5-monooxygenase activation protein, eta polypeptide | ENSMMUT00000013886 |  | 738 | 2 | Vpr; inhibits |
| ZC3HAV1 | zinc finger CCCH-type, antiviral 1 | ENSMMUT00000009178 |  | 2727 | 14 | Retropepsin; cleaves |
| ZAP70 | zeta-chain (TCR) associated protein kinase 70kDa | ENSMMUT00000030321 |  | 1863 | 12 | Pr55(Gag); regulated by |
| ZNRD1 | zinc ribbon domain containing 1 | ENSMMUT00000016967 |  | 378 | 4 | HIV-1 viral replication is enhanced by knock-down of human gene |

**Legend of Supplementary Table S2:**

*Macaca mulatta* sequences from the Ensemble database were used as references to create a sequence capture system. For several genes absent from the *Macaca mulatta* genome annotation, we used the human sequences as references. The interaction of genes with HIV-1 is detailed in the human interaction database (<https://www.ncbi.nlm.nih.gov/genome/viruses/retroviruses/hiv-1/interactions/>) ^2^.

**Supplementary Table S3: Sequence read information**

| Sample ID | Draft read numbers | Draft read bases (base number) | Average read length (base number) | Average quality value | Mapping summary | |
| --- | --- | --- | --- | --- | --- | --- |
|  |  |  |  |  | Coverage (%) | Average depth |
| 9204 | 1,841,059 | 238,797,052 | 129.7 | 23.6 | 97.7 | 102.1 |
| 11137 | 1,685,914 | 193,000,634 | 114.5 | 24.2 | 97.3 | 100.9 |
| 11245 | 1,943,618 | 243,539,031 | 125.3 | 23.7 | 97.7 | 102.0 |
| Z857 | 2,038,305 | 243,825,909 | 119.6 | 23.8 | 97.4 | 100.0 |
| 10435 | 2,388,279 | 309,699,208 | 129.7 | 23.9 | 97.7 | 132.7 |
| 10465 | 7,028,167 | 870,659,927 | 123.9 | 23.3 | 100.0 | 86.3 |
| Total | 16,925,342 | 2,099,521,761 | 742.7 | 142.6 | 587.7 | 624.1 |
| Average | 2,820,890 | 349,920,294 | 123.8 | 23.8 | 98.0 | 104.0 |
| ±SD | 1,893,789 | 235,348,199 | 5.4 | 0.3 | 0.9 | 14.0 |
| Maximum value | 7,028,167 | 870,659,927 | 129.7 | 24.2 | 100.0 | 132.7 |
| Minimum value | 1,685,914 | 193,000,634 | 114.5 | 23.3 | 97.3 | 86.3 |

**Supplementary Table S4: List of primers used for genotyping 12 SNPs by PCR followed by Sanger sequencing**

| Locus | Forward primer nucleotide (5’-3’) | Reverse primer nucleotide (5’-3’) | Amplicon size (bp) |
| --- | --- | --- | --- |
| CXCR2 | TGCTATGAGGACATGGGCAAC | TGTGCCCTGAAGAAGAGCCA | 481 |
| LEF1 | CCCCTCTGTCTTTCCTGCTGT | TTAAGGGCACGTTTTCCTGC | 594 |
| IFIT3 | ATTGGGTGCTGCTACAAGGC | CTGCCATCCTCAAGCTCAGAT | 560 |
| DDX53 | CCACAGAAGAAGAAAAACGAGCTC | CGTGTTTCTGTGTCCCTCTTTTGT | 505 |
| CD244 | GAGCACTGGAATTGGAGCCA | CCTCCACCCATTCATGTCTGA | 509 |
| EIF2AK3 | GGAGGTAGGCAGGTAGAAGAGGAC | GCCCCAAACTTCTTAATATCAGCA | 501 |
| IL18RAP | GCCCCAACTACGTCAACGG | TGCTGGCAGCAGACTCTGAAT | 474 |
| IL37 | TTGAAGGAGTGACAGGCAGGA | CCACCTTATGTGCCTGGACAC | 508^(1)^ |
| IFNGR1 | CCGCAGTAGCTGTGGGTTG | TGTACCGCCTGTCACTGCAC | 551 |
| IRF9 | TCAGGCTCTGAGAGCAGCATG | CGAGACTCTTGTGCTGGGCT | 502 |
| STAU2 | TTCCACACATCAGTTTCCCTTG | GCTTCAGCTGTAGAAGATGTTCCG | 502 |
| RANBP2 | GCCACAGCATCAAATCAGG | CATGAACTGCAATCCCACTGT | 719 |

**Legend of Supplementary Table S4:**

^(1)^ In the case of IL37, the length of amplified fragments is variable (508 or 535bp) due to the presence or absence of a 27 bp duplicated motif. This length variation was confirmed by using a nested PCR with the following primers: forward primer (5’-3’): IL37-exon1-F2 [5’ CAACGTTGAAAATGTCCTTTGTGG 3’] and reverse primer (5’-3’): IL37-exon1-R2 [5’ CCTGCTCTAGGAATTACACTTTGG 3’].

**Supplementary Table S5: Description of the four IL-37 NS-SNPs studied in humans**

| dbSNP rs# cluster id | Chr.2 position | Heterozygosity | MAF | mRNA pos ^(1)^ | dbSNP allele | Impact on protein | Protein residue | Codon pos | Amino acid pos ^(2)^ |
| --- | --- | --- | --- | --- | --- | --- | --- | --- | --- |
| rs3811046 | 112913801 | 0,464 | 0.3659 | 134 | T/G | missense | Val / Gly | 2 | 31 |
| rs3811047 | 112913833 | 0,457 | 0.3535 | 166 | G/A | missense | Ala / Thr | 1 | 42 |
| rs2708947 | 112918642 | 0,139 | 0.0753 | 532 | C/T | missense | Arg / Trp | 1 | 164 |
| rs2723192 | 112918804 | 0,139 | 0.0753 | 694 | A/G | missense | Asn / Asp | 1 | 218 |

**Legend of Supplementary Table S5:**

^(1)^ Position is given on IL-37 transcript variant 1 (sequence NM_014439).

^(2)^ Position is given on IL-37 isoform 1 (sequence NP_055254), also referred to as IL-37b.

These four SNPs are sufficient to define the most frequent IL-37 alleles observed in European and African populations ^3^. Based on their data, Kang et al. ^3^ recommended genotyping any one of the five non-synonymous SNPs (rs2708943:C>G, rs2723183: A>G, rs2723187:C>T, rs2708947:C>T or rs2723192:A>G) to differentiate between IL-37 haplogroups 1 and 2, which differ at a minimum of five non-synonymous sites and could result in varied susceptibility to human diseases. We explored two of the five recommended SNPs (rs2708947:C4T and rs2723192:A4G), thereby ensuring that we have differentiated between the two IL-37 main haplogroups.

**Supplementary Table S6: Multiple linear regression model taking into account the three SNPs candidate characterized in this study, eight previously characterized candidate genes ^1^, and two MHC class IB haplotypes.**

| Gene/region | Location of SNPs on chromosome ^(1)^ | Reference | Best linear regression model (probability of association, ANOVA) ^(2)^ |
| --- | --- | --- | --- |
| **CD244** | Chr.1: 90,806,827 | this study | **1.93 x 10^-2^** |
| IRF9 | Chr.7: 87,945,244 | this study | not associated |
| **IL37** | Chr.13: 17,064,452 | this study | **2.13 x 10^-4^** |
| UMODL1 | Chr.3: 4,684,432 | previous study ^(3)^ | not associated |
| SLC26A8 | Chr.4: 134,811,277 | previous study ^(3)^ | not associated |
| LOC102130113 ^(4)^ | Chr.5: 163,236,012  Chr.5: 163,262,889  Chr.5: 163,263,047  Chr.5: 163,263,341 | previous study ^(3)^ | not associated |
| MYH8 and MYH13 ^(5)^ | Chr.16: 10,573,814  Chr.16: 10,498,978 | previous study ^(3)^ | not associated |
| **MAGED4** (LOC102139876) | Chr.X: 50,696,215 | previous study ^(3)^ | **2.30 x 10^-3^** |
| **MHC class IB haplotype M2** | Chr.4 MHC class-IB region | previous study ^(3)^ | **1.50 x 10^-5^** |
| **MHC class IB haplotype M6** | Chr.4 MHC class-IB region | previous study ^(3)^ | **3.53 x 10^-6^** |

**Legend of Supplementary Table S6:**

Using a multiple linear regression (MLR) model, we tested the combined impact of the SNPs lying on the IL37 exon 1 and the eight good candidate genes (SNPs with probabilities of association <0.001) characterized previously ^1^, as well as two MHC class IB haplotypes on the control of PVL. Both forward and backward stepwise regression yielded the same model with five significant predictors, namely the M2 and M6 MHC haplotypes, IL37, MAGED4 and CD244. These five markers in conjunction explained 67% of the logPVL variance (*p* = 7.7 x 10^-9^). This linear model emphasizes again the probability of association of the IL37 NS-SNP.

^(1)^ The positions are given by reference to the assembly of the *Macaca fascicularis* genome MacFas5.0 (GCA 000364345.1).

^(2)^ We selected the best model by means of stepwise regression. Both forward and backward stepwise regression yielded the same model with five significant predictors, namely the M2 and M6 MHC haplotypes, IL37, MAGED4 and CD244. These five markers in conjunction explained 67% of the log PVL variance (p= 7.7x10^-9^).

^(3)^ Best candidates characterized previously ^1^.

^(4)^ The genotypes of the 42 animals were strictly homologous (base of reference / variant) for the four candidate SNPs in LOC102130113. We took only one SNP into account in the linear model regression.

^(5)^ The genotypes of the 42 animals were strictly homologous (base of reference / variant) for the SNPs in the MYH8 and MYH13 genes. We took only one SNP into account in the linear model regression.

**Supplementary Table S7: Polymorphic positions in the region surrounding IL-37 exon 1 in 44 cynomolgus Mauritian macaques.**

| Position *Mafa* Chr.13  (*Mafa* 5.0 Whole genome Annotation) | 17064239 | 17064328 | 17064347 | **17064414** | **17064452** | **17064494** | **i-13** | **i-21** | 17064525 | 17064616 | 17064617 | 17064618 | 17064630 | 17064642 |
| --- | --- | --- | --- | --- | --- | --- | --- | --- | --- | --- | --- | --- | --- | --- |
| most frequent (F) | G (0.84) | G (0.875) | G (0.875) | G (0.875) | C (0.83) | C (0.77) | C (0.76) | C (0.786) | C (0.77) | T (0.82) | T (0.825) | T (0.82) | G (0.814) | A (0.515) |
| variant (F) | A (0.16) | A (0.125) | A (0.125) | T (0.125) | T (0.17) | T (0.23) | T (0.24) | T (0.214) | T (0.23) | C (0.18) | A (0.175) | C (0.18) | T (0.186) | G (0.485) |
| animal# | 37 | 44 | 44 | 44 | 44 | 42 | 42 | 42 | 41 | 41 | 40 | 41 | 35 | 33 |

**Legend of Supplementary Table S7:**

The genomic sequences of exon 1 were studied from position 17064198 to 17064701 of *Mafa* chromosome 13. In most animals, we observed a 27 bp insertion by means of comparison to the *Mafa* whole genome annotation. This insertion corresponds to the duplication of a 27 bp motif. The length of the analyzed sequences was 531 bp. Numbering of positions is given by reference to *Mafa* whole genome annotation 5.0. Positions i-13 and i-21 correspond to the 13^th^ and 21^st^ positions of the 27 base repeat which is absent from the *Mafa* whole genome annotation and from rare cynomolgus alleles studied here (two animals had rare alleles without the 27 bp duplication).

Some sequences were incomplete. For this reason, we indicate the number of animals for which we obtained conclusive results for each variable position. The most frequent bases are identical to those observed in *Mafa* 5.0 whole genome annotation. The SNPs located in the coding region of exon-1 are underlined. The double underlined position (*Mafa* genome 5.0, Chr.13: 17064452) corresponds to the NS SNP (C->T; Thr->Met) which was associated with the set point PVL. The SNP located in position Chr.13 “17064414” is also non-synonymous (G->T; Leu->Phe). These two NS-SNPs are not linked. No SNP was fully linked to the SNP at position Chr.13: 17064239. The SNP that was most linked to the latter was located at position Chr.13: 17064452 (three animals among the 37 genotyped for Chr.13: 17064239 and Chr.13: 17064452 displayed discordant genotypes for the two latter SNPs).

**Supplementary Table S8**: **Location of Mauritius SNPs in the IL-37 coding sequence.**

| position on *Mafa* chr:13 | 11111111111111111111111  77777777777777777777777  00000000000000000000000  66666666666666667777777  44444588999999990000000  44444255000111990000111  15699378489246581345007  42214885496778724525090 |
| --- | --- |
| position on the cDNA coding sequence | 1122333444445555666  25690756378135682446017  19981585052334380181656 |
| exon number | 11111344555555666666666 |
| S/N | NNNNNNSNSSSNSSNSNNNSSSN |
| #9204  Low PVL | GCAACTRGGGTCCTATTCTAAAG |
| #11137 | K.....A................ |
| #11245 | ......G................ |
| #Z857 | .Y....G................ |
| #10435  High PVL | .Y....G................ |
| #10465_2 | .T..................... |
| #10465_1 | ....................... |
| #8102_cDNA | .T....G...........C.... |
| #8141_1_cDNA | ......A.........A...... |
| #8141_2_cDNA | ......A................ |
| #OBHJ6_cDNA | ......G...........C.... |
| #Ref_*Mafa* | ......A.........A...... |
| #Ref_*Mamu* | ......A..AC.T.......... |
| #*Hosa*_NM_014439 | -TGGTAACA.CA.CGA.T.GGG. |

**Legend of Supplementary Table S8:**

Positions on the coding sequence are given by reference to the *Mafa* coding sequence characterized in animal #9204, which has the 27 bp motif duplication in exon 1.

Ref_Mafa and Ref_Mamu sequences correspond to XM_015433396 and XM_002808051, respectively.

**Supplementary Table S9: SNPs reported in NCBI databank in the *Macaca mulatta* IL-37 gene.**

| Codon | Pos in the codon | Nt position | Nucleotides | | Amino acids | |
| --- | --- | --- | --- | --- | --- | --- |
|  |  |  | Ref ^(1)^ | Var | Ref ^(2)^ | Var |
| 15 | 1 | 43 | A | C | N | H |
| 18 | 1 | 52 | G | T | V | L |
| 20 | 2 | 59 | C | T | T | M |
| 56 | 3 | 168 | A | G | synonymous | |
| 65 | 2 | 194 | C | T | P | L |
| 73 | 1 | 217 | C | T | Q | STOP |
| 79 | 3 | 237 | C | T | synonymous | |
| 90 | 2 | 269 | A | G | D | G |
| 95 | 1 | 283 | C | T | R | C |
| 101 | 3 | 303 | G | A | synonymous | |
| 114 | 2 | 341 | G | A | G | E |
| 116 | 3 | 348 | A | G | synonymous | |
| 119 | 1 | 355 | C | T | synonymous | |
| 136 | 1 | 406 | T | C | STOP ^(3)^ | Q |
| 143 | 1 | 427 | T | A | L | M |
| 160 | 1 | 478 | C | T | R | W |
| 167 | 2 | 500 | C | T | A | V |
| 190 | 3 | 570 | T | C | synonymous | |
| 197 | 1 | 589 | G | A | V | M |
| 225 | 3 | 675 | C | T | synonymous | |

**Legend of Supplementary Table S9:**

The polymorphism in the *Macaca mulatta* IL37 coding sequence (*Mamu*) comes from the NIH database (https://www.ncbi.nlm.nih.gov/SNP/snp_ref.cgi?locusId=700579). The codon numbers and nucleotide positions are given by reference to the coding sequence XM_002808051 (the “A” of the start codon being position 1). The NS-SNP at codon 20 corresponds to the *Mafa* NS-SNP (Chr.13: 17,064,452) associated with controlling SIV infection in the cynomolgus macaque model (this study).

^(1)^ IL-37 nucleotide coding reference sequence*:* XM_002808051.

^(2)^ *Mamu* IL-37 protein reference sequence: (XP_002808097).

^(3)^ The *Mamu* IL-37 predicted mRNA sequence (XM_002808051) present a nonsense mutation at codon 136 (which corresponds to position 145 of *Mafa* IL37 protein in Figure 2, since the *Mamu* reference sequence lack the 27 bp repeat in exon 1). Another *Mamu* IL-37 predicted amino acid sequence (EHH22430; locus tag: EGK_05691) does not have a stop codon at position 144 and is 8 amino acids shorter at the amino-terminal extremity ^4^..

**Supplementary Table S10: Genotypes of the best three NS-SNPs using 22 animals above the median log-PVL and 22 animals below the median log-PVL.**

| **Locus name** | | **CD244** | **IRF9** | **IL-37 (IL1F7)** | **IL-36G 3’UTR** | **IL-36G 3’UTR** |
| --- | --- | --- | --- | --- | --- | --- |
| **Chromosome** | | chr1 | chr7 | chr13 | chr13 | chr13 |
| **location** | | 90,806,827 | 87,945,244 | 17,064,452 | 17,133,365 | 17,133,758 |
| **Ref./Var.** | | C/T | A/G | C/T | A/G | A/G |
| **Odd Ratio** | | 4.59 | 1.34 | 19.52 | **8.9** | **11.4** |
| **95% confidence** | | 1.27 to 21.09 | 0.51 to 3.57 | 2.70 to 863.87 | **1.773 - 63.019** | **1.928 - 127.17** |
| **Animals (route) [logPVL]** | ***p*** | 0.016 | 0.656 | 3.33 x 10^-4^ | **3.2 x 10^-3^** | **2.0 x 10^-3^** |
|  |  |  |  |  |  |  |
| 23037 (IR) [0,000] |  | C/C | A/A | C/C | A/A | A/A |
| 14468 (IR) [1,732] |  | C/C | A/A | C/C | nt | nt |
| 9204 (IV) [1,778] |  | C/C | A/A | C/C | A/A | A/A |
| 11137 (IV) [1,778] |  | C/C | A/A | C/C | A/A | A/A |
| 11245 (IV) [1,778] |  | C/C | A/A | C/C | A/A | A/A |
| 11637 (IV) [1,778] |  | C/T | G/G | C/C | A/A | A/A |
| 15885 (IR) [1,959] |  | C/T | A/A | C/C | A/A | A/A |
| OBHJ6 (IR) [2,153] |  | C/C | A/A | C/C | A/A | A/A |
| 9413 (IV) [2,222] |  | C/C | G/G | C/T | A/G | A/G |
| OBG7 (IR) [2,228]  Low PVL |  | C/C | A/A | C/C | A/A | A/A |
| OBPR6 (IR) [2,283] |  | C/C | A/A | C/C | A/A | A/A |
| Z776 (IV) [2,477] |  | C/C | G/G | C/C | A/A | A/A |
| 10515 (IV) [2,526] |  | C/C | A/G | C/C | A/A | A/A |
| 10228 (IV) [2,565] |  | C/C | G/G | C/C | A/G | A/G |
| 15461 (IR) [2,613] |  | C/C | A/A | C/C | A/A | A/A |
| 11296 (IV) [2,771] |  | C/C | A/G | C/C | A/A | A/A |
| OBFE6 (IR) [2,899] |  | C/C | A/G | C/C | A/A | A/A |
| 9691 (IV) [2,905] |  | C/C | A/A | C/C | A/A | A/A |
| 20595 (IV) [2,964] |  | C/C | A/A | C/C | A/A | A/A |
| 8249 (IV) [2,998] |  | C/C | A/A | C/C | A/G | A/A |
| 23060 (IR) [3,094] |  | C/T | A/G | C/C | A/A | A/A |
| 20351 (IR) [3,094] |  | C/T | G/G | C/C | A/A | A/A |
| 20654 (IV) [3,137] |  | C/C | A/A | C/C | A/A | A/A |
| 15232 (IR) [3,273] |  | T/T | A/G | C/T | A/G | A/G |
| 10116 (IV) [3,398] |  | C/C | A/A | C/C | A/A | A/A |
| 15693 (IV) [3,428] |  | C/C | A/A | C/T | A/G | A/G |
| 473 (IV) [3,499] |  | T/T | A/G | C/C | A/A | A/A |
| 10024 (IV) [3,544] |  | C/T | A/A | C/C | A/A | A/A |
| 9345 (IV) [3,614] |  | C/C | A/A | C/C | A/A | A/A |
| 20525 (IV) [3,925] |  | C/C | A/G | C/T | G/G | A/G |
| 9859 (IV) [4,004] |  | C/C | A/A | C/T | A/G | A/G |
| 8141 (IV) [4,053] |  | C/C | A/G | C/C | A/G | A/A |
| 15596 (IV) [4,053]  High PVL |  | C/T | A/A | C/T | A/G | A/G |
| 23014 (IR) [4,124] |  | C/C | A/A | C/T | A/G | A/G |
| OBRG6 (IR) [4,262] |  | C/C | A/G | C/C | A/A | A/A |
| 4763 (IV) [4,326] |  | C/C | G/G | C/C | A/A | A/A |
| 8102 (IV) [4,506] |  | C/C | G/G | T/T | G/G | G/G |
| 20483 (IV) [4,586] |  | C/C | A/G | C/T | nt | nt |
| 11360 (IV) [4,828] |  | T/T | G/G | C/C | A/A | A/A |
| Z860 (IV) [4,968] |  | C/T | A/A | T/T | G/G | G/G |
| Z857 (IV) [5,239] |  | C/T | G/G | C/T | A/G | A/G |
| OBRF6 (IR) [5,265] |  | T/T | A/A | C/C | A/A | A/A |
| 10435 (IV) [5,399] |  | C/T | G/G | C/T | A/G | A/G |
| 10465 (IV) [5,559] |  | C/T | A/G | C/T | A/G | A/G |

**Legend of Supplementary Table S10:**

A comparison to two candidate SNPs previously characterized in the 3’UTR region of IL-36G (De Manuel et al. 2018). Forty-two animals were genotyped for IL-37 SNP (17,064,452) and two SNPs in the 3’UTR region of IL36-G. Only one animal (#10228) is discordant in the comparison IL-37 SNP (17,064,452)/IL-36G SNP (17,133,758). Four animals (#10228, #20525, #8249, and #8141) are discordant in the comparison IL-37 SNP (17,064,452)/IL-36G SNP (17,133,365).

**Supplementary Figure S1:**

motif 1 motif 2 motif 3 motif 4

11111111112222222222333333333344444 44444555555555566666666667777777

12345678901234567890123456789012345678901234 56789012345678901234567890123456

#9204 MSNNSTLKMSFVGENSGVKTGSEDWEKDEPQCYSEKDEPQCYSE------------------DPAGSPLEPGPSLPSMNFVHTSPKVKNLNPKK this study

L

#11137 ...... .....................................------------------................................ this study

F

#11245 ............................................------------------................................ this study

M

T

#Z857 ................... ........................------------------................................ this study

M

T

#10435 ................... ........................------------------................................ this study

#10465_1 ............................................------------------................................ this study

#10465_2 ...................M...............---------------------------................................ this study

#8102 ...................M........................------------------................................ this study

#8141_1 ............................................------------------................................ this study

#8141_2 ...................................---------------------------................................ this study

#OBHJ6 ............................................------------------................................ this study

#XP_011722814_Mane ...................M........................------------------................................ OWM (Mane)

#XP_015288882_Mafa ...................................---------------------------................................ OWM (Mafa)

#EHH55843_____Mafa --------...........................---------------------------N............................... OWM (Mafa)

#XP_002808097_Mamu ...................................---------------------------................................ OWM (Mamu)

#EHH22430_____Mamu --------...........................---------------------------................................ OWM (Mamu)

#Mamu variants ..............H..L.M...............---------------------------.............................L.. see legend (1)

#XM_012071577_Ceat --------...........M........................------------------..................A............. OWM (sooty mangabey)

#XP_023050692_Pite ...................M........................KDEPQCYSE---------.............T.................. OWM colobe

#XP_017746967_Rhbi ...................M........................KDEPQCYSE---------.............T.................. OWM colobe

#XP_021780905_Paan .....A.............M........................KDEPQCYSE---------.L.............................. OWM baboon

#XP_008006593_Chsa ...................M........................KDEPQCYSDKDEPQCYLE............F.....A............. OWM vervet

#XP_010374651_Rhro --------...........M............CLTA--------------------------.............T.................. OWM colobe

#XP_011814860_Coan --------...........M............CLTA--------------------------.............T.................. OWM colobe

#NP_055254____Hosa --------...........M............CL.---------------------------..............T................. Hosa

#variant_4 --------...........M............CL.---------------------------..............T................. Hosa

#variant_9 --------...........M............CL.---------------------------..............T................. Hosa

#variant_3 --------...........M............CL.---------------------------..............A................. Hosa

#variant_8 --------...........MD...........CL.---------------------------..............T................. Hosa

#variant_1 --------...........M............CL.---------------------------...V..........A................. Hosa

#variant_13 --------...........M............CL.---------------------------...V..........A................. Hosa

#variant_7 --------...........M............CL.---------------------------...V..........A................. Hosa

#variant_12 --------...........M............CL.---------------------------...V..........T................. Hosa

#variant_11 --------...........M............CL.---------------------------.L............T................. Hosa

#variant_5 --------...........M............CL.---------------------------...V...-......A................. Hosa

#variant_2 --------...........M............CL.---------------------------..............T.......R...S..... Hosa

#variant_6 --------...........M............CL.---------------------------..............T.......R...S..... Hosa

#variant_10 --------...........M............CL.---------------------------..............A.......R...S..... Hosa

#XP_004031672_Gogo --------...........M............CL.---------------------------..............T................. Gorilla

#XP_003775916_Poab --------...........M............CL.---------------------------.L............A................. Orangutan

#XP_003277735_Nole --------...........M............CL.---------------------------..............AT................ Gibbon

#XP_003942551_Sabo --------....E......M....L.R.....C..---------------------------.........D.....TS...S...L....... NWM saimiri

#XP_012294372_Aona --------....E......M....L.R.....C..---------------------------.........D......S...S...L....L.. NWM aotus

#XM_003735278_Caja --------....E......M....L.R.....L..---------------------------GKLEA....D....F.S...S----------- NWM marmoset

#XP_008055305_Casy -------------......MN........F..C.G---------------------------.Q...F.......Y.VGSA.S.S.L.CKE... Prosimian (tarsier)

#XP_012512219_Prco --------..SLE...E..MD....G....W.C..---------------------------..........L.V..V.SA.S.A.LNARA.E. Prosimian (lemur)

#XP_012598282_Mimu --------..LLE......MDA...GE...W.C..---------------------------...R......L.V..V.S.PS.A..NARA.E. Prosimian (mouse lemur)

#Otga --------..LLE......MDY.N--------------------------------------..SR..P.S.L.CAHSGQ.NAHTQ.NPGA.QQ Prosimian (galago) (2)

**Legend of Supplementary Figure S1: Comparison of the amino-terminal region of the IL-37 proteins in humans, macaques, and other nonhuman primates.**

In most Old-World monkey sequences, there is a threonine at amino acid position 20. The variant associated with the PVL value described in the present study encodes a methionine, as in the human sequence. The non-synonymous SNP responsible for this amino acid change is shared by *Macaca mulatta* (see sequence “Mamu variants” and note 1 hereafter). Note that the number of nine amino acids motif 25-35 is variable from species to another. It is duplicated in most cynomolgus macaque alleles. In the present study, only two animals out of 44 (MAUR_6_10465 and 8141) have an allele (sequences MAUR_6_10465_2 and 8141_2) without this duplicated motif. The motif is also duplicated in *Macaca nemestrina* but not in *Macaca mulatta*. It is triplicated in two species of colobus (*Piliocolobus tephrosceles, Rhinopithecus bieti*) and baboon (*Papio anubis*). It is quadruplicated in *Chlorocebus sabaeus* (vervet). Note that the number of caspase 1 cleavage sites (KD/EP) varies from 1 to 4 as a function of the number of 9 amino acid motif repeats in the sequences.

Note 1: The “Mamu variants” sequence does not exist in databases. It is a compilation of all the amino acid variants found in the NCBI database (see SNP linked to Gene (ID:700579) Via Contig Annotation; <https://www.ncbi.nlm.nih.gov/SNP/snp_ref.cgi?locusId=700579>).

Note 2: In the *Otolemur garnetii* sequence, there is an insertion of one amino acid (after position 61) which was ignored in the alignment (CAHSGSQNAHTQ instead of CAHSGQNAHTQ).

Note 3: Significance of abbreviated species names.

Note 4: The degree of homology (% of identical amino acids) between the human IL37 (complete sequence of IL37b) and its nonhuman primate homologues varied from 97.3 to 99.1 for apes, 94.3 to 96.5 for Old World monkeys, 83.3 to 88.6 for New World monkeys, 62.7 to 70.6 for prosimians. A Neighbor-joining tree of IL37 protein sequences is shown in **Supplementary Figure S2.**

Note 5: Abbreviated species names are given in the table hereafter.

| Abbreviated species name | Species name | Vernacular suborder or parvorder | Taxonomy |
| --- | --- | --- | --- |
| Aona | *Aotus nancymaae* | NWM | *Platyrrhini; Aotidae* |
| Caja | *Callithrix jacchus* | NWM | *Platyrrhini; Cebidae; Callitrichinae* |
| Casy | *Carlito syrichta* | Tarsier | *Haplorrhini; Tarsiiformes; Tarsiidae* |
| Ceat | *Cercocebus atys* | OWM | *Catarrhini; Cercopithecidae; Cercopithecinae* |
| Chsa | *Chlorocebus sabaeus* | OWM | *Catarrhini; Cercopithecoidea; Cercopithecidae; Cercopithecinae* |
| Coan | *Colobus angolensis palliatus* | OWM | *Catarrhini; Cercopithecoidea; Cercopithecidae; Colobinae; Colobus* |
| Gogo | *Gorilla gorilla gorilla* | anthropoid ape | *Catarrhini; Hominoidea; Hominidae; Homininae; Gorilla* |
| Hosa | *Homo sapiens* | homosapiens | *Catarrhini; Hominoidea; Hominidae; Homininae* |
| Mafa | *Macaca fascicularis* | OWM | *Catarrhini; Cercopithecoidea; Cercopithecidae; Cercopithecinae* |
| Mamu | *Macaca mulatta* | OWM | *Catarrhini; Cercopithecoidea; Cercopithecidae; Cercopithecinae* |
| Mane | *Macaca nemestrina* | OWM | *Catarrhini; Cercopithecoidea; Cercopithecidae; Cercopithecinae* |
| Mimu | *Microcebus murinus* | prosimian | *Strepsirrhini; Lemuriformes; Cheirogaleidae; Microcebus* |
| Nole | *Nomascus leucogenys* | lesser ape | *Catarrhini; Hominoidea; Hylobatidae* |
| Paan | *Papio anubis* | OWM | *Catarrhini; Cercopithecoidea; Cercopithecidae; Cercopithecinae* |
| Pite | *Piliocolobus tephrosceles* | OWM | *Catarrhini; Cercopithecoidea; Cercopithecidae; Colobinae* |
| Poab | *Pongo abelii* | anthropoid ape | *Catarrhini; Hominoidea; Hominidae; Ponginae* |
| Prco | *Propithecus coquereli* | prosimian | *Strepsirrhini; Lemuriformes; Indriidae* |
| Rhbi | *Rhinopithecus bieti* | OWM | *Catarrhini; Cercopithecoidea; Cercopithecidae; Colobinae;* |
| Rhro | *Rhinopithecus roxellana* | OWM | *Catarrhini; Cercopithecoidea; Cercopithecidae; Colobinae* |
| Sabo | *Saimiri boliviensis boliviensis* | NWM | *Platyrrhini; Cebidae; Saimiriinae; Saimiri; Saimiri boliviensis* |
| Ceat | *Cercocebus atys* | OWM | *Catarrhini; Cercopithecoidea; Cercopithecidae; Cercopithecinae* |

**Supplementary figure S2:** Neighbor joining tree of IL37 protein sequences in nonhuman primates.

**Legend of Supplementary Figure S2**:

Neighbor joining tree of full length IL37 protein sequences.

The scale is given in number of amino acid differences. For more details see note 4 in the legend of **Supplementary figure S1.** For significance of abbreviated species names, see note 5 in the legend of **Supplementary figure S1**.

**Supplementary figure S3: IL37 relative expression levels before and after SIV inoculation**


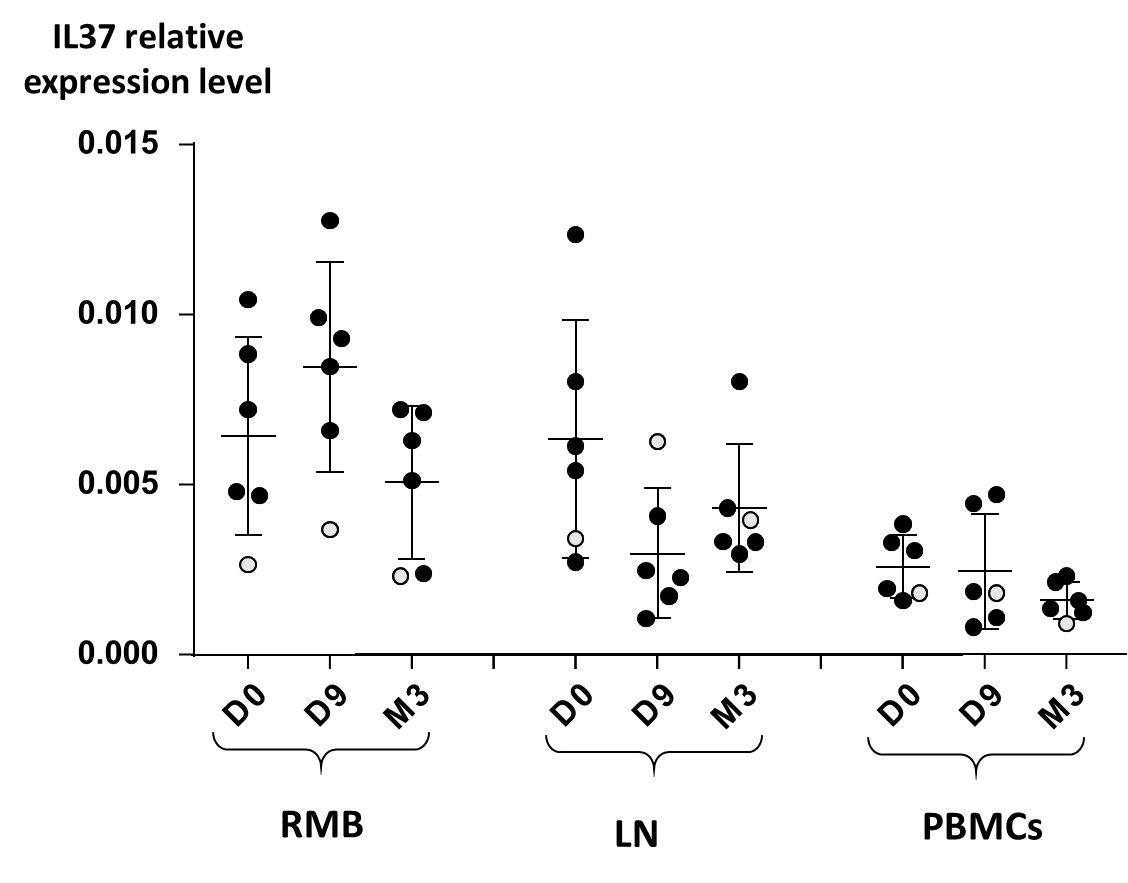


**Legend Supplementary figure S3:** Labeled RNA samples were hybridized to Agilent rhesus macaque *Macaca Mulatta* custom 8*60K array (AMADID 045743, Agilent technologies). For more details see materials and methods and Echebli et al. 2018 ^5^. For each sample, IL37 expression level is expressed relative to GAPDH levels. The relative expression level of IL37 was studied in various tissues (rectal mucosae biopsy: RMB, peripheral lymph node: PLN, peripheral blood mononuclear cells: PBMCs) of six animals inoculated intravenously with 5,000 AID50 of SIVmac251. The IL37 relative expression levels are given prior to inoculation (D0) and nine days (D9) or three months (M3) after inoculation. All animals were genotyped for the IL37 NS SNP associated with control of SIV infection. One animal (circle colored in grey) was heterozygous for this SNP, while the other five were homozygous.

**Supplementary figure S4:** Correlation between the PVL at the set point and the fold-change of IL-37 relative expression at D9 in the rectal mucosae (RMB).


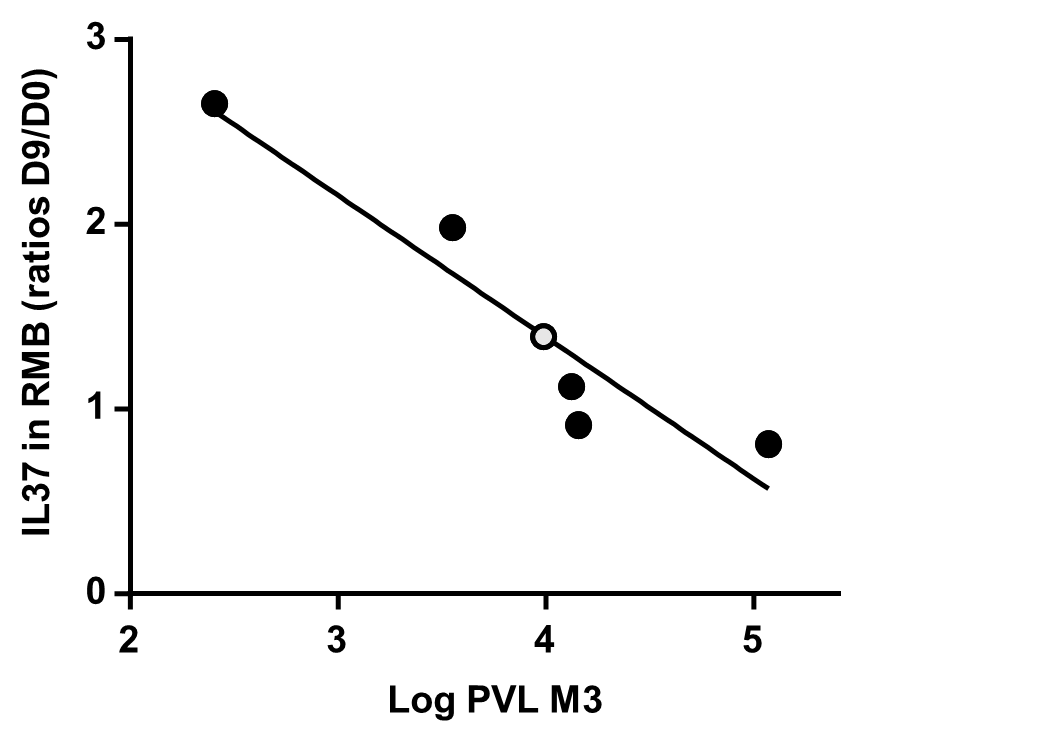


**Legend Supplementary figure S4:** Relative expression levels of IL37 in various tissues were obtained as described in **Supplementary figure S3** legend. A significant negative correlation between the PVL at the set point and the fold-change of IL-37 expression at D9 RMB (R² = 0.89, p=0.0047 Pearson’s test ; r_s_^2^=1, p=0.0027 Spearman’s test). In contrast, fold-changes in IL37 expression in RMB at M3 or in PBMCs and PLNs on D9 and M3 did not correlate with the PVL at the set point.

**Supplementary Figure S3:** Comparison of CD244 exon 9 genomic sequences in Mauritian macaques to macaque sequences in database.

Mafa Ch1:90806827 Mafa Ch1:90806848

🡩 🡩

[ 111 111 111 122 222 222 223 333 333 333 444 444 444 455 555 555 556 666 666 666 777 777 777 788 ]

[ 123 456 789 012 345 678 901 234 567 890 123 456 789 012 345 678 901 234 567 890 123 456 789 012 345 678 901 ]

#allele-1 ATT GAA AAG AGT CAA CCT AAA GCC CAG AAC CCT GCT CGA CTG AGC CGC AAA GAG CTG AAG AAC TTT GAT GTT TAT TCC TAG

#allele-2 ... ... ... ... ... ... ... ... ... ... ... ... T.. ... ... ... ... ... ... G.. ... ... ... ... ... ... ...

#Mafa2 ... ... ... ... ... ... ... ... ... ... ... ... T.. ... ... ... ... ... ... G.. ... ... ... ... ... ... ...

#Mafa1 ... ... ... ... ... ... ... ... ... ... ... ... ... ... ... T.. ... ... ... G.. ... ... ... ... ... ... ...

#Mafa3 ... ... ... ... ... ... ... ... ... ... ... ... ... ... ... ... ... ... ... ... ... ... ... ... ... ... ...

#Mane ... ... ... ... ... ... ... ... ... ... ... ... ... ... ... ... ... ... ... ... ... ... ... ... ... ... ...

#Mamu ... ... ... ... ... ... ... ... ... ... ... ... ... ... ... ... ... ... ... ... ... ... ... ... ... ... ...

#Hosa ... .G. ... ... ... ... ... ... ... ... ... ... ... T.. ... ... ... ... ... G.. ... ... ... ... ... ... ...

[ 1 1 1 1 1 1 1 1 1 1 2 2 2 2 2 2 2 2 ]

[ 1 2 3 4 5 6 7 8 9 0 1 2 3 4 5 6 7 8 9 0 1 2 3 4 5 6 7 ]

#allele-1 I E K S Q P K A Q N P A R L S R K E L K N F D V Y S *

#allele-2 . . . . . . . . . . . . * - - - - - - - - - - - - - -

#Mafa2 . . . . . . . . . . . . * - - - - - - - - - - - - - -

#Mafa1 . . . . . . . . . . . . . . . C . . . E . . . . . . .

#Mafa3 . . . . . . . . . . . . . . . . . . . . . . . . . . .

#Mane . . . . . . . . . . . . . . . . . . . . . . . . . . .

#Mamu . . . . . . . . . . . . . . . . . . . . . . . . . . .

#Hosa . G . . . . . . . . . . . . . . . . . E . . . . . . .

**Legend of Supplementary Figure S3:**

Comparison of CD244 exon 9 genomic sequences in Mauritian macaques to macaque sequences in database.

Allele-1 and allele-2 were from the present study.

The Mafa1 sequence (LT160000) is from a Mauritian cynomolgus macaque genome annotation ^6^.

The Mafa2 sequence (XM_015454750, XM_015454744, XM_015454740, XM_015454739, XM_015454735) is identical with the Mauritius allele 2. It is deduced from the *Macaca fascicularis* 5.0 Ref. Seq. assembly [GCF_000364345.1].

The Mafa3 sequence (CM001276) is from the *Macaca fascicularis* whole genome shotgun sequence ^4^. It is identical to Mauritius allele-1.

Mane (XM_024798084, XM_011770106, XM_011770105, XM_011770104) and Mamu (XM_015113655, XM_015113652) sequences are from annotations of the *M. mulatta* and *M. nemestrina* genomes, respectively.

The Hosa sequence (NM_016382) corresponds to a human mRNA sequence (splicing variant 1).

**References**

1 de Manuel, M. *et al.* Whole genome sequencing in the search for genes associated with the control of SIV infection in the Mauritian macaque model. *Scientific reports* **8**, 7131, doi:10.1038/s41598-018-25071-x (2018).

2 Ako-Adjei, D. *et al.* HIV-1, human interaction database: current status and new features. *Nucleic Acids Res* **43**, D566-D570, doi:10.1093/nar/gku1126 (2015).

3 Kang, B., Cheng, S., Peng, J., Yan, J. & Zhang, S. Interleukin-37 gene variants segregated anciently coexist during hominid evolution. *European journal of human genetics : EJHG* **23**, 1392-1398, doi:10.1038/ejhg.2014.302 (2015).

4 Yan, G. *et al.* Genome sequencing and comparison of two nonhuman primate animal models, the cynomolgus and Chinese rhesus macaques. *Nature biotechnology* **29**, 1019-1023, doi:10.1038/nbt.1992 (2011).

5 Echebli, N. *et al.* Stage-specific IFN-induced and IFN gene expression reveal convergence of type I and type II IFN and highlight their role in both acute and chronic stage of pathogenic SIV infection. *PloS one* **13**, e0190334, doi:10.1371/journal.pone.0190334 (2018).

6 Ebeling, M. et al. Genome-based analysis of the nonhuman primate Macaca fascicularis as a model for drug safety assessment. Genome Res 21, 1746-1756, doi:10.1101/gr.123117.111 (2011).
